# Cancer Stem Cells and the Tumor Microenvironment: Targeting the Critical Crosstalk through Nanocarrier Systems

**DOI:** 10.1007/s12015-022-10426-9

**Published:** 2022-07-25

**Authors:** Aadya Nayak, Neerada Meenakshi Warrier, Praveen Kumar

**Affiliations:** Department of Biotechnology, Manipal Institute of Technology, Manipal Academy of Higher Education, Manipal, 576104 Karnataka India

**Keywords:** Nanocarrier Targeting, Cancer Stem Cells, Tumor Microenvironments, Stemness Pathways, Stemness Biomarkers, Cancer Signaling

## Abstract

The physiological state of the tumor microenvironment (TME) plays a central role in cancer development due to multiple universal features that transcend heterogeneity and niche specifications, like promoting cancer progression and metastasis. As a result of their preponderant involvement in tumor growth and maintenance through several microsystemic alterations, including hypoxia, oxidative stress, and acidosis, TMEs make for ideal targets in both diagnostic and therapeutic ventures. Correspondingly, methodologies to target TMEs have been investigated this past decade as stratagems of significant potential in the genre of focused cancer treatment. Within targeted oncotherapy, nanomedical derivates—nanocarriers (NCs) especially—have emerged to present notable prospects in enhancing targeting specificity. Yet, one major issue in the application of NCs in microenvironmental directed therapy is that TMEs are too broad a spectrum of targeting possibilities for these carriers to be effectively employed. However, cancer stem cells (CSCs) might portend a solution to the above conundrum: aside from being quite heavily invested in tumorigenesis and therapeutic resistance, CSCs also show self-renewal and fluid clonogenic properties that often define specific TME niches. Further scrutiny of the relationship between CSCs and TMEs also points towards mechanisms that underly tumoral characteristics of metastasis, malignancy, and even resistance. This review summarizes recent advances in NC-enabled targeting of CSCs for more holistic strikes against TMEs and discusses both the current challenges that hinder the clinical application of these strategies as well as the avenues that can further CSC-targeting initiatives.

**Graphical abstract:**

Central role of CSCs in regulation of cellular components within the TME

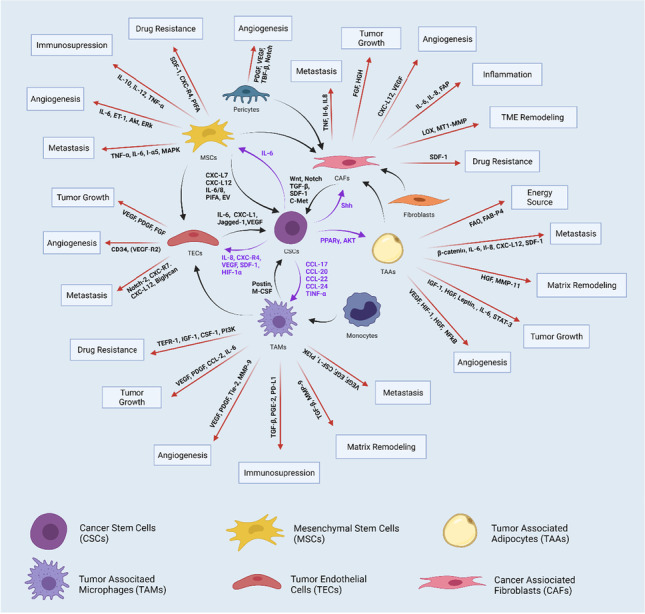

## Introduction

### Cancer Stem Cells in the Tumor Microenvironment

There is often a misconstrued perception of cancer as a singular, unitary mass when it is in fact an organ system of sorts, within which cells are recruited for transformation into malignancy. The interaction network that builds around these cells is what constitutes a tumor microenvironment (TME) and has a large variety of cells, both malignant and non-malignant, that act as nodes within this network, including endothelial cells, pericytes, myeloid cells, mesenchymal stem cells, immune cells and fibroblasts [1–3]. The extracellular matrix (ECM) is also heavily involved, with significant research pointing towards the ECM playing a critical role in intratumoral signaling, transportation, and immunogenicity within malignant tissue, solid tumors to be specific [4, 5]. The TME, in essence, is the cellular environment that is based around tumors or cancer stem cells (CSCs) and is responsible for the progression of the cancer within its host system, predominantly through its support for hyperproliferation.

Stem cells, on account of their tumor regenerative properties and their participation in tumorigenesis in terms of initiation and metastasis, are a key component of the TME for tumor progression [6, 7]. Cancer-associated fibroblasts (CAFs), one of the differentiated end-products of CSCs, are at the forefront of this tumor system-remodeling that aims to improve proliferative capacity, higher plasticity, and even drug resistance. CAFs manage to accomplish the remodeling as mentioned above via intercellular adhesion molecules (ICAMs) and cytokines like hepatocyte growth factors (HGFs), epidermal growth factors (EGFs) and interleukin-6 (IL-6) that promote cancer cell survival and proliferation while simultaneously pushing for progression of the tissue into metastasis [7–9].

One of the major reasons the TME is under such limelight when it comes to focusing target therapy is its role in tumorigenesis—a property attributed to it predominantly due to CSCs. In addition to generalized cancer progression, CSCs also induce traits of drug resistance and regenerative capacity in tumor cells that are the primary causes of ineffective clinical trials for cancer [6, 10, 11]. Furthermore, CSCs regulation is heavily ingrained in interaction with their corresponding microenvironments. Research points towards cancer cells getting triggered into displaying resistance and other stem cell-like properties as a result of certain environmental conditions [12]. Such trials have been used to identify environmental markers that bring about the unwelcome traits of resistance and tumor progression in oncotherapy which allows for treatments to be more directed and niche-specific in their approach.

CSCs have been unquestionably established as playing a central role in the setbacks currently faced in clinical trials and pre-clinical research. Thus, devising a system that can target them at both a cellular and systemic level within the TME is the most promising of the presented avenues in the evolution of therapeutic design. By reviewing nanoparticular drug delivery systems (DDSs) targeting a variety of CSC niches that present genuine potential in clinical implementation, this paper aims to both address the shortcomings in current DDS designs when it comes to CSC targeting and provide a scaffold on which a multi-fronted format of cancer therapy can be supported.

### Nanomedicine and its Applications in Cancer Therapeutics

Nanobiotechnology may have various applications in other fields of science, but some of its most significant applications remain within the pharmaceutical and biomedical sciences, where current study has significantly progressed conventional systems of drug delivery [13, 14]. As a field, nanomedicine has progressed so far from its roots of a novel application in the current stream of therapeutics that the US National Cancer Institute (NCI) was able to withdraw funding for the Center of Cancer Nanotechnology Excellence (CCNE), confident that the field was well enough established to be self-standing [15].

While its most promising domain of application remains cancer, nanomedicine also shows a great deal of potential in varying fields of medicine: their ability to pass through the blood–brain barrier (BBB) via either transcytosis or endocytosis allows nanomedicines to implement highly efficient treatment to the central nervous system (CNS) and any of the diseases that plague it. There are several preclinical trials of nanomedicine with animal models of brain diseases, including gliomas [16], Huntington's [17], and even Alzheimer's [18], with a particular focus on transcytosis, which enables passage of not just smaller molecules but also nucleic acids and proteins, in a non-invasive manner [19].

Reverting to oncotherapeutics, the predominant effort in the ongoing battle against cancer has always been in its eradication, in complete cure. However, it is just as important to consider the process from the patient's perspective. The current regimen of chemotherapy, immunotherapy, and radiation is painful and invasive, and it makes the road to recovery an extremely uncomfortable one. While complete cure will always be the ultimate goal, improved life quality of the patients on the receiving end of this treatment is also a matter that needs to be urgently addressed [20]. This therapeutic sector is where nanomedicine shows the greatest promise—in non-invasive reorganization of the current regime [21].

As for the nature of its applications, nanomedicine forks into two approaches on how it can enable improvisations in current cancer therapy: it can either create an entirely new drug to target cancer in a highly specific manner or better the specificity of current delivery models [18, 19]. The focus of this review will be on the improved administration of pre-existing drugs.

### Carriers in Nanomedicine for Drug Targeting

Nanoparticles (NPs), on account of their size and unique properties (volume-to-charge ratio), act as a link between bulk matter and its composite molecular and atomic structures. Some of the major contributions of nanobiotechnology within medicine are in disease diagnosis and target-specific drug delivery [11]. Therapeutic approaches include drug delivery where NPs can either be applied as therapeutic particles or as casings for the intended drug. They are typically involved in tissue and cell level interactions, and their biggest application is as carriers of active drugs in drug delivery models so as to ensure specific release of the active drug and its extended maintenance within the patient's system [22–24].

These nanocarriers (NCs) are particularly advantageous in the medical field because a large degree of the scopes currently employed for detection technology development trigger the body's immune response [23, 25, 26]. Consequently, drug targeting is an especially important application when it comes to cancer cell systems. Widespread treatment regimens (chemotherapy, immunotherapy) are designed to kill cells in the tumor vicinity and thus, run the risk of harming or altering healthy cells in the process. To overcome this issue, drug specificity to the tumor cells becomes paramount. An important advantage of NC employment is that it is non-invasive while also being capable of accessing deeper tissues and can be precisely controlled and focused onto specific target sites [27, 28].

Extending beyond just drug administration, NCs also present potential for in vivo long-term tracing systems specific to CSCs, most popularly in the form of metallic NPs [29–31] and fluorescently labelled aggregation-induced emission (AIE) dots [32]. Such applications, while important as preventive measures against secondary cancers borne from metastasis, also allow for closer insight on the details of the role and interactions of CSCs within the TME.

### TME and CSCs in Cancer Metastasis and Drug Resistance

It is now a well-established fact that cancer is significantly harder to treat once it begins metastasizing, with almost 90% of cancer deaths being accounted to metastatic tumors as compared to primary tumors [33]. Not only does the TME play an integral role in the progression of a cancerous cell into metastasis, but the change to a TME once the tumor is settled at a secondary site is also a major factor to ponder when answering the question of why metastatic tumors are far more lethal than their primary brethren [34].

### Microenvironmental Involvement in Metastasis

The 'how' of TME involvement in cancer metastasis is now relatively well-established. A plethora of TME cells, including CAFs, immune-inflammatory cells, adipose cells, and neuroendocrine cells (NECs), interact with the blood and lymphatic networks to create a self-propagating system of excessive proliferation, tumorigenesis, and metastatic growth. This network in turn is regulated by a large number of cytokines and chemokines, including but not limited to platelet-derived growth factors (PDGFs), vascular endothelial growth factors (VEGF), fibroblast growth factors (FGFs), transformation growth factors (TGFs) and their corresponding receptors [35–37]. While the TME does differ vastly depending on the cellular histology and the location of a tumor mass, the most cardinal—and by extension, the most common—TME cells are CAFs like adipocytes [38 39], myofibroblasts, and mesenchymal stem cells (MSCs) [9, 40, 41]; immune-inflammatory cells like regulatory T (Treg) lymphocytes, B lymphocytes, tumor-associated macrophages (TAMs), myeloid-derived suppressor cells (MDSCs) [42, 43] and neutrophils [36]; angiogenic vascular cells like pericytes [44]; and other miscellaneous cells like NECs [8] and dendritic cells (DCs) [3, 42]. Given their involvement in several cancer hallmarks, these cells then form the basis of primary targeting employed in all the current forms of cancer therapy, depending on their functional influence pertaining to a particular form of cancer.

**Fig. 1:**
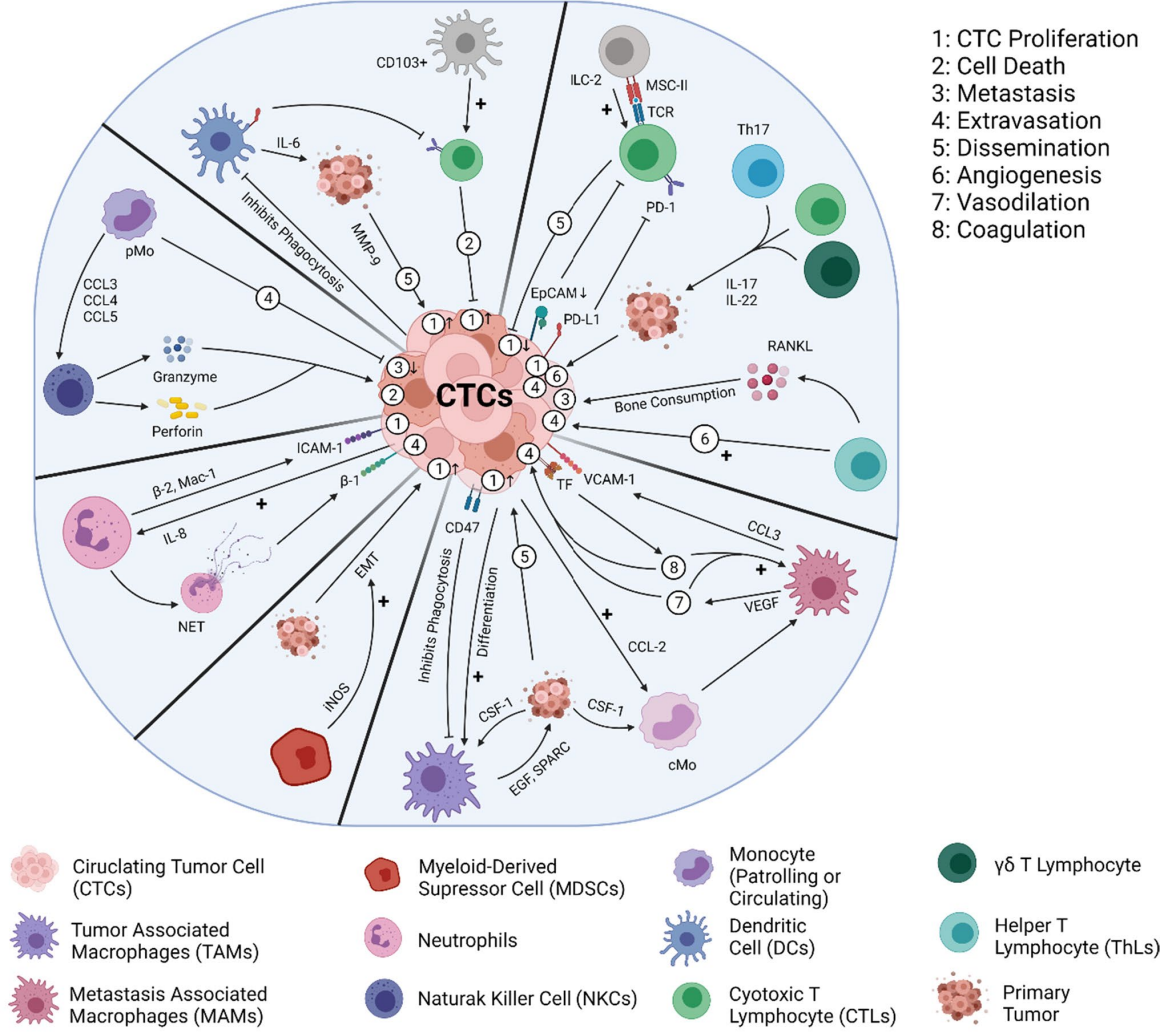
CTC interactions during the metastatic process. The above figure details the varying interactions that CTCs undergo, alongside their potential contributions towards the differentiation of the secondary TME from its pre-metastatic state. Out of these interactions, those with the immune aspects of the circulatory system seem to have the greatest impact on the TME’s characterizations between primary and secondary metastases [49]. (Created with BioRender)

Aside from considering the TME's effect on cancer progression, the differences between metastatic and primary TMEs, as well as their corresponding influence on cancer invasion, must also be taken into account [45–47]. Their perceived role in the aggressive nature of metastasized cancer is yet another reason TMEs should be viewed as ideal targets in cancer therapeutics. Following the Paget theory of 'seed and soil' in 1889 for metastatic spread, it has been an increasingly circulating notion that the TMEs of metastases are bound to be distinct from that of their 'seed' (the primary tumor) despite both the cells being of the same histological origins. While the environment of the secondary site does play a role in this differentiation, there is also involvement of the interactions a circulating tumor cell (CTC) undergoes when migrating towards a parenchymal site among distant tissues [34, 36]. Upon intravasating into the blood stream as either individual cells or multicell clusters, CTCs’ interactions with neutrophils and platelets become a means of progressing tumor metastasis as they respectively facilitate extravasation and prevent both tumor cell recognition as well as lysis from NK cells [36, 48]. Other interactions involve macrophages, MDSCs, and lymphocytes and cumulatively converge around the final goal of CTC invasion and the establishment of a secondary site [34, 48–50], as compiled within (Fig. 1).

A cross-cancer comparison of primary and metastatic ovarian tumors was able to profile some characteristics that distinguished the two corresponding TMEs. On account of metastatic growth, tumor cells developed a higher density of TME cells with disease progression and resulted in better regulation of malignant-cell derived chemokine and cytokine networks (with IL-16 playing a crucial role in their orchestration) [51]. There was also an increased concentration and alignment of collagen bundles within the neighboring ECM, as well as a close correlation between tissue stiffness—and by extension, cellular rigidity for better survival—and disease progression. The same index was also cross-referenced against other cancers and definitively concluded that ECM-associated gene expression in connection to the matric index was applicable across all human cancers [52]. Aside from this, a clinical study on luminal breast cancer differentiation between primary and metastatic sites validated the immune component of the TME cells mentioned in the above review by establishing the bearings for a potential bridge between metastatic TMEs and cancer relapse [47]. It insinuated that verified lower tumor-infiltrating lymphocyte (TIL) concentration at metastases, due to increased cell density at the secondary site, could be linked to a reduced autoimmune response as well as other conditions encouraging overall survival and relapse [47, 51, 53, 54].

The TME's involvement in all aspects of tumor growth and progression, extending as far as relapse, makes it the optimal target when considering prognosis. However, with directed treatment arises the issue of TME differentiation between cancer types and sub-types, which would require painstaking studies and profiling of TMEs for all the varied cancers and their corresponding metastases at different parenchyma. While this method is wholly valid and effective, a more efficient approach would be to identify and isolate a keystone element within the network that would afford a greater wield of control over the TME as a whole—and this is where CSCs come to play.

### Stem Cells as a Point of Origin for Cancer-Associated Tumor Microenvironment Cells

There are many brands of thought on the origins of TME cells; the two most common are that these cells originate from the neighboring tissues [55] or that they originate from cancer cells [6]. While the most likely model is a hybrid of the above two approaches, the focus of this paper is on CSCs. They are a self-renewing cell type responsible for the maintenance and proliferation of tumor tissue as well as metastatic initiation, and can even differentiate into various TME components through stem cell pathways, like Notch, Hedgehog, Wnt, and TGF-β [56–58].

As such, there are predominantly two models for CSC contribution to tumorigenesis—namely the classical model and the plasticity model [59–63]. In the context of the former, variegated cell phenotypes in a TME are primarily the result of CSC differentiation after a microenvironmental alteration [59, 62]. The plasticity model builds on the foundation of the classical model and addresses the interchangeable conversion between differentiated adult cells and CSCs. While CSC differentiation into non-CSCs is well characterized via stem cell pathways, stemness transcription factors like Oct-3/4, Klf-4, Sox-2, PI3K [64, 65] as well as epigenetic regulations like DNA methylation/demethylation at CpG islands, histone modifications, nucleosome positioning in correspondence to the above genes [66] also help revert an adult cell into induced pluripotent stem cells (iPSCs) [67, 68]—thus the model’s nomenclature. The interchangeable maintenance between CSCs and non-CSCs within the TME suggested by the plasticity model goes a long way in explaining CSC robustness, as it likens to the benefits of genetic variation for a population’s survival initially proposed in Darwin’s theory of evolution [61, 62, 68].

Both these models prescribe to the overarching hierarchical model of tumorigenesis, which assumes a progenitor between adult stem cells and actual tumor heterogeneity in CSCs [59, 62, 63]. However, most cell populations within the TME seem to adhere to the stochastic model of random mutagenetic accumulation [57, 60, 62]. Here, heterogenic tumor cells have been hypothesized to be induced from chronic inflammation or from conditions wherein normal stem cells or progenitor cells are induced through mutagenesis to become cancerous [7, 59]. Currently, in clinical conditions, iPSCs hold the most potential in the direct generation of CSCs [59]. CSCs derived from iPSCs reprogrammed from normal cell lines could differentiate into multiple tumor components, including CAFs, TAMs, adipocytes, and tumor-associated endothelial cells (TECs) [7]. While the pathways of CSC differentiation aren't entirely mapped out and are bound to be questioned for validity, there is definitive proof that CSCs can differentiate into CAFs [7, 69]. CAF involvement in tumorigenesis, cancer progression, and drug resistance by induced heterogeneity [70–73] alone should be sufficient reason to seriously consider CSCs as a target in oncotherapy, especially considering how stromal cell targeting has already shown results in overcoming chemoresistance [74, 75]. A review of the graphical abstract would better highlight the (quite literal) central role that CSCs have in TME-supported cancer progression, which in turn marks them as 'keystone' targets, capable of bringing the entire tumorigenesis pathway to a stalemate if hit successfully.

### Stem Cell Involvement in Tumor Microenvironment Regulation

In addition to being a core contributor to the various cellular sub-populations that comprise the TME, CSCs are also heavily involved in the regulation of the microenvironment via a plethora of their characteristics. Some of the core behaviors that characterize CSCs are deregulated hyperproliferation, resistance to cell death, hypoxic autophagy, ferroptosis, increased angiogenesis, and increased induction of metastasis [58, 76–78]. CSCs attain excessive levels of self-renewal through participation in stemness pathways, including Hedgehog [79–82], Notch [83–87], Wnt/β-catenin [83, 88, 89], Nanog [90–92], NF-kB [93, 94], RAS [95, 96], p38 MAPK [97–100], PI3K [100–102], and EGFR pathways [103–105]. As has been elaborated, CSC participation in these pathways is key to its differentiative capacity and overexpressed stem pathways can be associated with biomass growth in tumors [58, 106, 107].

Increased vasculature is another characteristic of CSCs that involves heavy cross-activity with the TME [8, 108, 109]. Angiogenesis is an important part of microenvironmental maintenance and also plays central role in immune evasion, hyperproliferation, metastasis, and therapeutic resistance [5, 110–116]. Moreover, CSC-initiated angiogenesis via factors like VEGF, Ang-2, MIP-2, TGF-β1, IL-6, and IL-8 as well as vasculogenic mimicry are also regulated by stemness pathways [111, 117–119]. Angiogenetic factors like the von Willebrand factor [120–123], Tspan-8 [124, 125], the chemokines CXCL5 [126–129] and MIF [130–133], the CCR chemokine receptor family [50, 134–136] are often mediated through exosomes to ensure vascular up flux and endothelial regeneration [111–113, 137]. The ECM, a core element of the TME, also plays a critical role in determining proliferative tendencies during angiogenesis as well as regulating CSC differentiative capacity by impacting cellular stiffness [4, 5, 114, 138].

One of the most impactful ways CSCs interact with the neighboring TME is through their modulation of hypoxia [51, 57, 139, 140]. The result of a cellular proliferation rate that can no longer be supported by the transfer rate of oxygen from blood, hypoxia’s key presentations include suppressed apoptosis [141–144], progression of EMT [144–148], malignancy and distant tumor metastasis [141, 142, 149–152], and deregulated angiogenesis [139, 153–155]. Hypoxia regulates on the basis of hypoxia-inducing factors (HIFs) and their interactions with stemness pathways, transcriptions factors, and other cancerous agents [152, 155, 156]. Pillar interactions include HIF-1α with TGF-β1 via a SMAD-dependent pathway [157–161] or with Notch-1 [162, 163] for hypoxic initiation of EMT, with VEGF [164–166] for angiogenic regulation, and with GLUT-1/3 [167, 168] and hexokinase (HK)-1/2 [169–172] for a shift towards glycolytic metabolism [169]. Other common associated markers include LOX [173–175], MMP [173–175], Twist [176, 177], STAT3/IL-6 [178–183], MAPK/ERK [184–186], Sox-2 [187–190], Oct-4 [191–193], and c-Myc [194–197]. While the HIF family remains the primary mediators of the hypoxic response, research also points towards exosomal involvement in various hypoxic functionalities [151, 198–201]. Given their vesicular nature, exosomes’ involvement in the TME does shine light on the possibility of their usage as next-gen NCs, specifically in the context of CSC-targeting.

The plasticity model clearly establishes a horizontal axis of differentiation between CSCs and non-CSCs, with the implication that inter-differentiation is the primary cause of cancerous cellular tendencies. Yet, there is evidence in traced lineages indicative of stochastic growth patterns in tumor tissue [202]. This indicates a sensitivity to the microenvironment suggestive of a feedback control loop in CSC maintenance [63, 65, 202]—in fact, TME regulation of CSC plasticity has even been linked to regulated quiescence [203, 204], which is central to immune escape [110, 205] and metastatic initiation in CSC and other tumor-initiating phenotypes [204, 206, 207]. Quiescence-induced tumorigenesis during immune-compromised conditions as well as the endowed immunosuppressive properties also contribute towards CSC-mediated chemoresistance [203–205, 208].

Given the highly involved interconnection between CSCs and the TME, the relevance of a multi-fronted targeting mechanism that can initiate anti-cancer activity at both a cellular and microenvironmental scale becomes significantly more promising in enabling non-recurrent cancer recovery.

### Stem Cell Heterogeneity and Drug Resistance

The CSC theory holds that tumor growth is fueled by specific stem cells. The corresponding model is also based off four key features: cellular heterogeneity, self-renewal, limited plasticity within tumor hierarchy, and drug resistance [209]. The multi-drug resistance phenomenon that currently plagues all cancer therapy is on account of CSCs, induced by endogenous detoxifying enzyme expression, higher levels of drug efflux, decreased drug response, hypoxic stress on the TME, or even increased DNA repair activity [31, 210–213]. The mechanism of CSC drug resistance is via stem cell pathways. They express ATP-binding cassette (ABC) transporters, which are multi-drug resistant and can eliminate potential for drug damage. Even if the cells undergo some degree of injury, certain CSC markers like stem pathways also help negate oxidative stress by removing free radicals and induce resistance to chemotherapeutic drugs. CSCs also activate DNA repair capabilities within tumor cells, which contributes towards protection against apoptotic factors [58, 209].

Cancer drug resistance at a tumor level is enforced predominantly through two phases of rejection—the tumor can either be intrinsically resistant or develop resistance through positive selection of an unaffected subpopulation [73, 212, 214]. Given CSC involvement in the cellular and biomolecular make-up of tumors from initiation to metastasis, they by default become the focus of therapeutic resistance: they contribute both the cancer-associated cells that characterize innate resistance as well as the heterogeneity and survival mechanisms in the form of stem cell pathways that ensure sustained tumor proliferation and evolution [215, 216]. These very mechanisms also go on to increment an eventual trigger for a relapse in the disease [216].

## Current Regimens for Cancer Therapy

Targeted delivery systems were developed primarily to address the need for regulated concentrations of drugs to be administered long-term. Initial systems were characterized by immediate release upon entry into the system. Thus, the compound would be partially or fully metabolized before it could reach the actual terminus, leading to both reduced efficacy of the treatment and risks of side-effects from metabolic by-product accumulation in non-related organelles [217]. Targeted delivery systems comprise the active drug being introduced directly into the organelle in question, with minimal widespread release. The drug's design, then, no longer has to bear in mind any interactions it will partake in before it reaches the target cell or tissue and can instead hone more towards amplifying anti-tumor activity.

### Cancer-Specific Drug Targeting Therapy

The two streams of targeted delivery have remained consistent over the past decade, with the major variations being limited to only the targeting mechanisms and the delivery vehicles. The principle of the delivery itself remains preserved. These two strategies are namely active and passive targeting systems.

Passive targeting in cancer is characterized by its use of the anatomical and functional differences between normal and tumor vasculature to ensure a selective accumulation of drugs at the tumor site, dependent on enhanced permeability and retention (EPR), impaired lymphatic drainage, and localized delivery [218]. The EPR effect enables smaller compounds to accumulate far easier in tumorous tissues than healthy ones due to the former's heterogeneous vascularity and highly permeable membrane: this in turn ensures a modicum of tumor-selectivity within the delivery mechanism so that minimal healthy cells are tampered with. Localized delivery, on the other hand, involves direct delivery of the drug to a specific tumor site to exclude the systemic side effects of the drugs while also concentrating drug levels at their site of action [218–220].

Active DDSs are designed upon the basis of specificity to either vascular endothelium or tumor cells by making use of affinity ligands. Endothelium cells are ideal targets as they are easily accessible through circulation, are genetically stable, and tend not to develop resistance against therapeutic agents. Further, they are easier to mark on account of the angiogenetic processes that they undergo, wherein the development of new blood vessels in tumor tissue to meet nutritional requirements results in activated endothelial cells that show elevated expression of adhesion molecules and proteolytic enzymes [221, 222]. In the case of tumor cells, several proteins are overexpressed in comparison to healthy cells and can serve as significant biomarkers for the progression of the disease and as surrogate markers for an indirect measure of drug therapy efficacy. These abovementioned biomarkers, preferentially expressed in cancer cells, are also known as tumor-associated antigens (TAA). Aside from TAA-based targeting, tumor cells are also an ideal locus of targeting given that they present cell-surface receptors (CSRs) to a higher degree for increased nutrition influx, which also makes for easier drug uptake. Aside from surface CD markers, the most commonly presented receptors to induce intake are folate receptors, LDL receptors, and hormone receptors [217, 220].

**Fig. 2:**
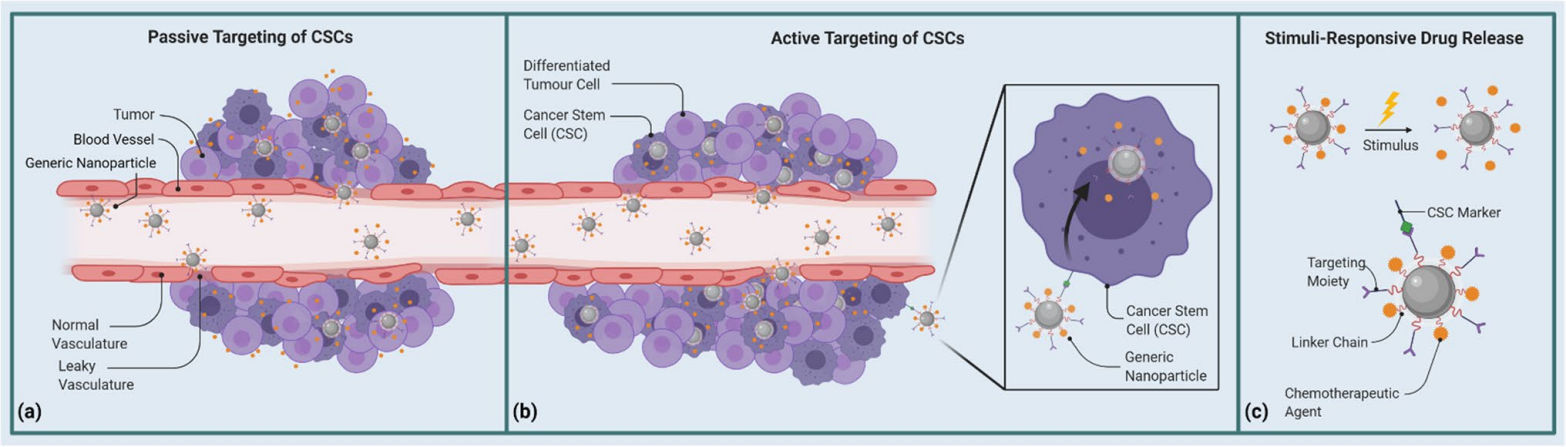
Principles of active and passive targeting for targeted drug delivery as well as stimuli-responsive drug release. **a** Passive targeting of CSCs, **b** Active targeting of CSCs, **c** Stimuli-responsive drug release. The above panel elaborates on the two main methodologies of targeted drug delivery to CSCs within tumors, namely active and passive targeting. It shows how passive targeting (a) is the basis over which active targeting (b) is a more specific overlayer. It approaches the tumor in the same manner as passive targeting, by taking advantage of the circulatory system and points of distorted endothelial lining near tumoral bases. But the actual biodistribution of the drug is intracytotic, made more direct to CSCs by engaging specifically with markers exclusive to particular CSC niches. This specificity is further enhanced by the mechanism of stimuli-responsive drug release (c), which caters to a spectrum of internal and external stimuli. (Created with BioRender)

One way of looking at these two targeting systems is as a sequence, as depicted in (Fig. 2). At its essence, the principles of active delivery ride on those of passive targeting: in both systems, the localization of the NC to the target tissue is through the circulatory system, by taking advantage of the 'leaky' vasculature—gaps in the endothelial lining of blood vessels that result from poorly controlled angiogenesis and subsequent EPR [217, 220, 223]. The difference is solely on the basis of specificity. Passive targeting uses EPR as its selective mechanism, which active targeting incorporates to further hone in on a tumor niche in particular. Between the two, active targeting is preferable for cancer-based applications simply because EPR isn't a selective enough factor to base the targeting of chemotherapeutic agents over. This is even more applicable in the case of metastatic malignancies that aren't established enough to be subject to EPR. A literary survey of NC-delivery to solid tumors spanning the past decade reported a median of 0.7% for the percentage of successful targeting [22]; even if this degree is significantly higher than the efficacy of free drug administration, at face value such a low degree of efficiency does bring into question the validity of the EPR effect as an efficient target.

A particular development in NCs as vehicles for active targeting is the concept of stimuli-responsive drug release. This is applicable in both passive and active mechanisms of targeting but serves its purpose better for active targeting applications. There is no control over the drug compound once it has been liberated, so having stimuli-responsive release doesn't necessarily contribute to the specificity of passive delivery. Because the CSC markers commonly targeted during active delivery are often involved in endocytic mechanisms [221, 222], the drug release in such contexts happens within the target cell, where no further control over the compound is necessary. Overall, their capacity to enable "on-demand" drug distribution that is spatiotemporally controlled makes these stimuli-responsive NCs an extremely attractive solution to the issue of premature release within encapsulated delivery models. Among such NCs, the "sensitive" aspect is typically attributed to the linker chain holding the chemotherapeutic agent to the main body of the carrier. The stimulus induces either protonation, hydrolytic cleavage, or a molecular conformational rearrangement of the linker [224], all of which essentially block the site adhered to the anticancer agent, thus resulting in the latter's biodistribution. As such, controlled release is a highly applied feature in NC-mediated drug delivery, in response to a plethora of stimuli both external and internal, including magnetic fields, electronic fields, heat, light, ultrasound for the former and pH, redox, hypoxia, enzyme activity for the latter [224, 225]. To make the stimuli-responsive system even more attuned to TME-specific interactions, a proposed multi-stimuli tactic specific to the internal stimuli set of hypoxia, enzymatic regulation, redox, pH, and ROS also holds a degree of popularity [226].

Nevertheless, it is something to note that despite targeted delivery showing immense success in pre-clinical studies, there has been very little turnover in these NCs being employed for clinical use. Even out of the ones that have made it past clinical trials, there is no incidence of an actively targeting NC [227]. One potential reason for such nominal biocompatibility can be the several physiological barriers that NCs are faced with, including endothelial barriers during extravasation, potential degradation from endocytic pathways, the escape of endocytosed NCs from the endo-lysosomal system because of vesicles, and even mononuclear phagocytic system (MPS) clearance, all of which significantly reduce the efficiency with which NCs can home their deliveries [223, 227]. These issues, however, can be bypassed via localized delivery, which essentially affords simultaneous control over the location of release, compound diffusion rates, and even duration and retention of both the release and the compound [227]. As for the low turnover rate of NCs, several meta-analyses of NC translation from in vitro to in vivo environments indicate as markers of successful biotransitions the fair correlation between the two models for hemolysis, coagulation, complement activation, opsonization, phagocytosis, immunosuppression, and thrombogenicity, albeit in the context of immunotoxicity alone [228]. But because there is no definitive correlation between in vivo and in vitro set-ups for the above factors, translating NC-based systems to in vivo environments becomes too unpredictable to be clinically viable. Other major issues include blood-incompatibility [229] and endotoxin contamination [230, 231], with the latter being liable for nearly 30% of the failed preclinical assessments by the US NP Characterization Laboratory [59].

Consequently, alternative means of targeting like TME modulation [232–234], and biological methods like cellular hitchhiking [235], extracellular vesicles [236–238], and even attenuated bacteria [239, 240] are also prominently being considered as potential solutions to the above issue of NC delivery [241].

### Cancer Stem Cell Targeting

CSCs are indisputably important to the progression and the severity of cancer; they are consequently very potent as targets for various cancer therapies. However, CSC heterogeneity makes it difficult to associate a cellular marker for a CSC niche. Furthermore, even if a more generic marker like CD44, CD133, or ALDH was to be targeted, these markers are often shared with normal stem cells, negating the primary advantage of high-specificity that is the premise of nanotherapy [56, 242]. Thus, the alternatives are to either target a group of cellular markers or the regulation of stemness pathways, or even a combination of both.

Because CSCs have such a high degree of heterogeneity, targeting an individual cell-surface receptor or marker often proves ineffective. Thus, the characterization of marker combinations specific to certain cancers is an integral part of successful drug targeting. The most universally common markers across the span of different cancers are CD44, CD133, EpCAM, and ALDH [6, 58, 76, 243]. These markers would be beneficial if used in the context of localized treatment; if the treatment is administered directly to the tumor, marker specificity is less of a concern and the emphasis is on therapy intake over accurate delivery. Alternatively, these markers are often used in tandem with another, more specific marker as a means of ensured uptake. The cancer-specific marker is dealt the responsibility of limiting the drug delivery to a specific CSC niche, whereas the more common marker makes sure the drug is without fail taken up by the cell—the more frequent cell markers often deal with metastasis and general cancer progression processes associated with stemness as compared to histology-specific properties, which is why targeting them is a fairly sure-shot means of assured drug administration [15, 21, 22].

While surface markers are an incredibly effective means of targeting CSCs, the degree of influence a treatment possesses depends directly on the efficacy of the drug compound and its successful intake by the cell. The system here works towards simply killing the root cause and doesn't take into consideration the interactome around the tumor cells that is responsible for the progression of the cancer. Given the recently developed role of epigenetics in stem cell differentiation [244, 245], transcription factors like Sox-2 [246, 247], Oct-4 [248, 249], Nanog [92, 250], CXC-R4 [244, 245], survivin (Birc5) [251–253], nestin [254], and Klf-4 [255] and their co-expression [255–258] are also promising avenues through which to capacitate efficacious therapy [91, 259]. When considering CSCs as the point of attack within the TME—wherein targeting CSCs via microenvironment subsections of hypoxia, vasculature, and cellular components such as TAMs or CAFs is prevalent [67, 260]—a far more potent approach would be to target the stem cell pathways that imbue these cells with the properties of plasticity, heterogeneity, and increased proliferation, which are answerable to ineffective treatment. The pathways most prominently associated with Wnt/β-catenin [89, 261–263], Notch [87, 264, 265], Hedgehog [81, 82, 266], NF-kB [267–269], JAK/STAT [270–274], PI3K/PTEN/AKT [102, 275], and PPAR [276, 277] pathways, all of which, in addition to the properties listed above, display common tendencies towards proliferation, tumorigenesis, metastasis, and survival, as well as secondary stem traits like drug resistance and self-renewal. By extension, the increased frequency of stemness pathways in breast, lung, liver, colon, and rectal cancers is an indicator that CSCs might also be playing a role in cancerous incidence. Statistically, cancers that have a higher degree of involvement from multiple stem cell pathways have an increased chance of emergence, simply because the higher replication rates of stem cells allow for increased mutagenic prevalence [278]. Targeting these pathways, then, is a direct parry on the defense that stem cell involvement provides regular tumor cells against current therapeutic protocols (Table 1).

**Table 1:** High-Frequency CSC Markers for Common Cancer Types

Cancer type	Breast	Colon	Glioma	Lung	Prostate	AML
Markers	CD44^+^/CD24^−^ ALDH1 CD90 α_6_-integrin Bcrp-1 IL-2 SDF-1 /CXCR4	CD133^+^ ESA + CD166 β-catenin LGR5 ABCG5 Survivin CD44^+^/EpCam	CD15 CD133 α_6_-integrin Nestin Sox-2 L1CAM SALL4 OLIG2	ABCG2 ALDH1 CD133 CD90 CD117 CD176	PSA ALDH1 CD44 CD133 α_6_-integrin α_2_/β_1_-integrin	CD34^+^/ CD38^−^

NC-based regimes have shown high efficacy against these internal and external biomarkers [279, 280] and have considerable potential in this particular application of oncotherapeutics. While combinational therapy in the sense of the loaded agent has been received with widespread applicative popularity, targeting multiple markers is limited by the marker location—as of such, combinational therapy of an internal biomarker and an external one together is yet to receive experimental consideration. Although there is insufficient scientific evidence, multi-level targeting can be argued to be more effective, especially when it comes to attacking CSCs, because it hinders both of their mechanisms of escaping apoptosis (self-renewal and multilineage differentiation) [83], thus improving chances of therapeutic results.

**Fig. 3:**
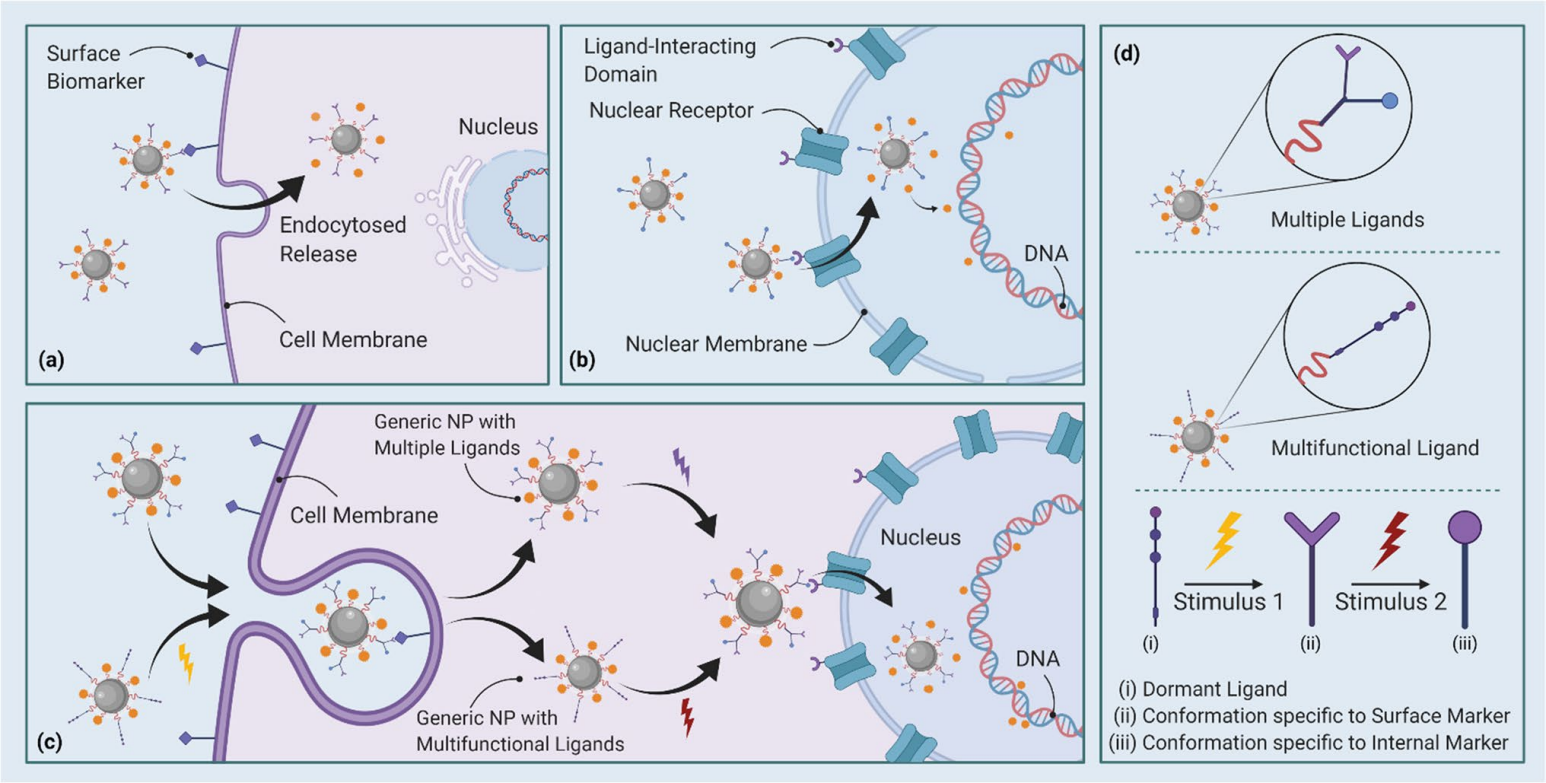
Model for multi-level targeting of CSCs via multiple or multifunctional ligands. **a** Targeting through surface biomarkers, **b** Targeting through ligand-interacting domain on the nuclear receptor, **c** Targeting through generic NP with multiple and multifunctional ligands, **d** Representation of multiple and multifunctional ligands. The above figure describes the basis through which prevalent NC-mediated targeting of CSCs via surface biomarkers (a), and genetic biomarkers (b), can be hypothetically merged into a model launching a multi-level attack (c). The model involves two ligand-orientation hypotheses, enabled through rounds of varying stimuli (d). The first is of a generic NP conjugated with two or more ligands that are respectively compatible with the external and internal markers being targeted. The functionalization of these ligands is a matter of steric organization and will differ in pertinence to the stem cell niche. The second model is of a multifunctional ligand that can be coaxed into different conformations compatible with specific levels of biomarkers, via rounds of distinct stimuli. (Created with BioRender)

An effective model, hypothetically, would be of an NC that can target CSCs both at the cellular and the genetic levels [140, 281]. As such, this would be possible through means of either a multifunctional ligand or multiple ligands that can be separately functionalized with different stimuli, as depicted within (Fig. 3). In the case of the former, the ligand's conformational changes in response to separate stimuli (ideally of different natures altogether) will enable specificity towards surface and core biomarkers individually. Unfortunately, this will require either the fortuitous discovery of a peptide sequence that is sensitive to a variety of environmental responses—with subsequent conformations compatible to a pair of common CSC biomarkers—or the synthetic design of a similar one. Both cases will require several rounds of design and optimization, entailing that such a ligand will not make an entry into the therapeutic market any time soon. As for the latter design of NCs conjugated with multiple ligands, the potential of immediate application is comparably higher. Both ligands can either be introduced dormant, with two separate stimuli to activate corresponding ligands, or with the ligand specific to the surface biomarker already functionalized. Having one pre-functionalized ligand improves the ease of design on several attributes: ligand sensitivity to the cellular microenvironments can be overlooked if it need not be activated; managing steric hindrance becomes easier as only one of the conjugates will be undergoing conformational changes towards functionality. However, this model must also contemplate how the functionalization of the second ligand will be affected by the conformation of its pre-functionalized companion—including an inspection of potential channels to inactivate or detach the same. While multifunctionalized NCs do show a great deal of promise in theory, effective optimization of ligand density, its effect on protein adsorption, as well as covalent attachment of the therapeutic agents to the functionalized NP for endocytosis and binding selectivity is still underdeveloped, forming an impediment in such NPs' widespread use [282, 283]. Moreover, they also mandate a real-time tracking system to ensure accurate drug disposal, which only further encumbers their realization.

### Nanocarrier-Based Cancer Therapeutics

Since its ideation, nanomedicine has consistently been a front runner for the next novel alternative in oncotherapy. To date, cancer remains the primary genre of NP-based clinical trials at 65%, despite a distinct increase of interest in other areas of application, including anesthesia, inflammation, and infection, over the past decade [21, 33]. NCs have proven extremely versatile in their involvement, even within the umbrella of 'cancer.' Not only do they cater to the severe variation within cancer histology, but they also allow for control on the nature of the therapeutic delivery.

Having concluded that CSCs are the most potent target for cancer-specific nanotherapy, we reviewed several clinical trials and case studies in hopes of narrowing down upon a wide-effect system with specifications for the nature of the NC, the most effective style of delivery as well as the CSC marker. While there are several ongoing and successfully completed clinical trials dealing with the nanoparticular administration of anti-cancer agents [284–295], the specific niche of CSC-targeted NCs remains to be clinically broached. The findings from the review, which consist primarily of pre-clinical in vitro models, have been organized within (Table. 2).

**Table 2:** Nanocarrier Systems for Various Cancer Stem Cells

Nanocarrier	Therapeutic Agent	Cancer Type	Delivery Model	Cell Line	CSC Marker	Cellular Uptake	Change in Lifespan	CSC Viability
Graphene oxides (GO)	-	Breast	GO flakes in 5% DMSO-distilled water dispersions in local targeting [296]	MCF-7	Wnt/β-catenin, Notch, NRF2, INFγ-STAT1	-	-	40%
	Salinomycin	Ovarian	RPE-rGO-Ag nanocomposite [297]	A-2780	ALDH1, CD133	-	-	15%
Dendrimers	siRNA	Brain	Anti-Lyn siRNA loaded onto phosphorous dendrimer [298]	BTSC-233, JHH-520, NCH-644	CD47, PD-L1, TIM3	80%	-	25%
Carbon nanotubes (CNT)	Salinomycin	Gastric	Chitosan-coated SWCNT activated by hyaluronic acid (SAL-SWCNT-CHI-HA) [299]	AGS	CD44^+^	21%	+ 30 d	11%
	Paclitaxel, Salinomycin	Breast	CD44 antibody hydrazone-linked onto SWCNT with pH activated release system [300]	MDA-MB-231	CD44^+^	50%	-	25%
Gold sphere nanoparticles (Au-NP)	siRNA	Breast	Au-NP conjugated with multiple units of glucose-polyion complexes linked with lipoic acid (Glu-PEG-PLL-LA) [301]	MDA-MB-231	GLUT1	35%	-	50%
	Salinomycin	Breast	PEGylated Au-NP (SH-PEG-NH_3_) [302]	MCF-7	CD24^−^, CD44^+^	63%	-	25%
	CD44v6 mAB	Gastric	PEGylated Au-NS conjugated with CD44v6 monoclonal antibody [303]	MKN-45	CD44	83% (non-specific)	+ 28 d	89.2%
	Teleglenastat	Brain	PEGylated Au-NP conjugated with CD133 aptamers loaded with teleglenastat (Au-PEG-CD133-CB-839) [304]	GBM-1, NCH-644	CD133	30%	-	50%
Gold nanorods (Au-NR)	Adriamycin	Liver	EpCAM antibody conjugated onto lipophilic Au-NR [305]	Hepa 1–6	EpCAM	27%	-	20%
	CXCR4 antibody	Gastric	Au-NRs conjugated with CXCR2 antibody (AuNR-SiO_2_-CXCR4) [306]	MGC-803	SDF1	75%	+ 21 d	40%
Liposomes	Paclitaxel, Salinomycin	Lung	AEYLR peptide-PEG-modified paclitaxel loaded nano-structured lipid carrier (NLC) [307]	NCI-H1299	CD133	95%	-	31.4%
	Docetaxel, Telmisartan (pre-treatment)	Lung	Docetaxel loaded PEGylated liposomes [308]	NCI-H460	CD133	96.4%	-	20%
	Curcumin-difluorinated (CDF)	Head and Neck	Liposomal CDF suspended in 0.9% NaCl, injected intravenously [242]	CCL-23R, UM-SCC-1R	CD44	-	-	45%
	Doxorubicin, Salinomycin	Liver	Redox-triggered dual-targeted liposome [309]	Huh-7	CD133, EpCAM, (Sox-2, Oct-4)	86%	-	11.8%
Polymeric nanoparticles (PNP)	Doxorubicin, Thioridazine	Breast	Both compounds loaded onto separate MTC-OBn polymer-ring micelles and delivered in-tandem [310]	BT-474, MCF-7	CD24^−^, CD44^+^	40%, 54%	-	20%
	Salinomycin, Docetaxel	Gastric	Both compounds loaded onto separate poly(D,L-LA-co-glycolic acid)-PEG PNPs but delivered in-tandem [311]	MKN-45, HMNI-N87	CD44^+^	80%	-	40%
	siRNA	Brain	PEG-PLA PNP loaded with FAM-siRNA [312]	U-251, U-87MG	GLUT3	60%	-	48%
	Naproxen	Breast	PNP coated with hyaluronic acid (HA-NP) [313]	MCF-7	CD44^+^, Cox	65%	-	45%
	miR-486	Lung	Cationic lipid core-crosslinked NPs (CCL-486) [314]	NCI-H460, NCI-A549, NCI-H1299	CD133, PI3K/AKT	78%	-	12.5%
	Paclitaxel	Lung	PGLA-PEG PNPs conjugated with CD133 aptamers [315]	HCC-827, A-549, A-431	ALDH, CD133	80%	-	40%
	Curcumin, Salinomycin	Breast	PEGylated PNPs conjugated with hyaluronic acid (HA-PEG-PLGA-Cur-Sal) [316]	MCF-7	CD24^−^, CD44^+^	96%	-	10%

Although there are several studies designed around the involvement of NCs in directed cancer therapy, most choose to target other aspects of the tumor biome over CSCs. The below table collates the most commonly used NCs targeting CSC markers, listing research with efficacies higher than 85%, determined on the basis of comparative CSC growth inhibition.

While NCs can be used in a variety of formats (drug carriers, direct therapeutic agents, passive carriers for antibodies), purely based on frequency, a general representation of the current upcoming nanomedicine regime for CSC therapeutics would be of a drug carrier (most likely a metal or polymeric NP or a liposome) ferrying salinomycin in combination with another anticancer drug [212, 299, 300, 307, 317]. In terms of sheer potency, drugs like paclitaxel and doxorubicin have an upper edge over salinomycin in tumor toxicity, as is explained by their frequent use in chemotherapy. However, their lack of specificity means that their administration runs the risk of several side effects, including myelosuppression, neurotoxicity, cardiovascular toxicity, gastrointestinal reaction, and hair loss [307].

Salinomycin, however, has very high specificity towards CSCs because it targets ABC-binding transporters, as well as the Wnt/β-catenin, Hedgehog, Notch, and Akt signaling pathways—thus ensuring a direct treatment for all the aspects of stemness that hinder cancer treatment [317, 318]. It also activates the p38 MAPK cascade which helps induce ROS-mediated apoptosis [319, 320]. Any combinational therapy with salinomycin consequently proves incredibly effective when it comes to CSC-specific therapy, as has been proven with in vitro trials. For instance, smart liposome-based systems co-delivering doxorubicin and salinomycin were found effective in reducing stemness in liver CSCs [309] whereas a combination of salinomycin and docetaxel loaded onto PNPs proved a promising strategy when targeting gastric CSCs [311]. Besides co-delivery systems, pre-clinical studies also show salinomycin derivates as capable of targeting CSCs successfully on their own, although predominantly within breast cancers models [321–323].

Despite its many promising properties in CSC targeting, administering salinomycin does come with certain obstacles—particularly in its aggressive hydrophilicity [318, 324]. This entails a dependency on nanodelivery, which can hinder its long-term relevance as issues of toxicity and systemic flushing continue to stand in the way of NC-based therapeutic systems circulating the market [26, 325]. Functional changes like conjugation with PEG or Vitamin E to form a prodrug can improve its solubility, but the design’s efficacy is acceptable only when employed within an NC-based format [324, 326, 327].

While not heavily scrutinized in this review, exosomes do present a promising alternative for NCs as they have already been characterized to be heavily involved in the crosstalk between CSCs and the TME and thus do not need to be additionally functionalized for specificity, and can also overcome biocompatibility issues that other inorganic DDSs are hindered by [279, 328–330].

It is also of notable import that a major portion of the current clinical trials that target CSCs are directed towards either breast or other solid tumor cancers. Despite there being no definitive proof hinting that nanotherapy has reduced effects on other cell types, the above trend can be used to hypothesize that NCs can target endothelial cells to a higher degree than other cell histologies [331].

As has been discussed, CSCs can be targeted via the two avenues of cellular markers or stemness pathways. Aside from delivering anticancer drugs, NCs can also be employed in a parallel system as vehicles for immunotherapy [332]. When considering cell surface markers, commonly used therapeutic agents include surface antigens (SAs) and immune checkpoint blockades (ICBs); alternatively, the aspects of immunotherapy engaged in pathway interactions include inhibitors for Wnt, Notch, Hedgehog, PI3K, and other metabolism or niche mechanisms [90, 265, 333]. While the focus of this paper is on the optimization of current NC-based systems in targeting stem cell markers and pathways, alternative CSC applications like CSCs as vehicles of delivery [238, 334, 335] or even infused stem therapy [336] cannot be dismissed. As such, MSCs in the context of therapeutic carriers are gaining rapid popularity as a strategy to ensure ameliorated side-effects on account of improved biocompatibility [332, 337].

## Conclusion

TMEs are a crucial aspect of cancer progression and play major roles in tumorigenesis, metastasis, and even relapse. Because they interact with almost all aspects of the tumor biome, TMEs can often be too large to successfully silence simply by blocking or competing against some of its constituent cells. In remediation however, CSCs prove to be ideal focal points for TME-directed targeting on account of their central role within TMEs. Common CSC markers across various niches include CD133, ALDH-1, CD44, and CD24, although there are several CSRs that are more niche-specific and thus better for drug delivery targeting. Furthermore, targeting common markers present significant limitations given the fact that they do not deliver in their promise of identifying all CSCs. Because stem cell populations amplify tendencies towards clonogenic and tumor heterogenic processes, common markers most often can't recognize more than specific cell subpopulations. Moreover, they're often also expressed on normal stem cell surfaces, which only reduces the efficiency of the targeting system. Thus, as a general modicum, common CSC markers are often used in combinational targeting, whereas more specific markers are focused upon for stand-alone targeting mechanisms.

While targeting systems have consistently been with CSRs in mind, if they are implemented toward stem cell pathways, they would be arguably more effective and even potentially overcome issues of MDR and relapse. There is a definitive turn in targeting systems toward stemness, but because common stem cell pathways like Wnt, Notch, and Hedgehog are also heavily involved in regular cell proliferation and maintenance systems, contained impairment of the pathway in a manner that doesn't bleed the effects onto neighboring cell biomes is yet to be conclusively defined. This is partly because much about the TME is yet to be uncovered. While there have been decisive leaps in the characterizations of several cellular and non-cellular components within microenvironments, their functions or signaling mechanisms are yet to be entirely chalked out. So far, the focus has been on major stromal and immune cell types and specific cell populations indigenous to particular stem niches. However, TME–CSC crosstalk in a physiologic context remains understudied. Pre-established organoid approaches towards cellular crosstalk have definitely improved in vitro modeling, but understanding at a microenvironment level of bio-nanointerfaces is essential for the further establishment of nanoparticular delivery systems.

Currently, organic NCs like liposomes or PNPs are particularly selected for, especially for more challenging target locations like brain tumors on account of higher biocompatibility. But as a whole, metallic NPs and carbon-based NPs are gaining wide-range popularity as mediums for drug targeting as well. The defining factor remains in how non-organic NPs need to undergo surface functionalization to mimic biocompatibility that their organic counterparts forego. Current protocol leans towards focused administration of pre-existing chemotherapeutics, perhaps in facilitation of response to disease urgency than for lack of scientific novelty. While this methodology has been producing steady results with improved drug efficacy and higher rates of recovery, it remains a fact that the current line of NC-based targeting systems isn't efficient enough to entirely keep highly toxic compounds like doxorubicin, paclitaxel, docetaxel, and temozolomide from leaking into the surrounding microenvironment. Aside from synthesizing a new drug altogether, an alternative could be to turn towards a different range of drugs, especially if the point is to target stemness. While salinomycin has become a commonly employed compound in such contexts for its stem-specific targeting, other polyether antibiotics can also be considered for similar applications.

As such, there is significant advancement in the delivery aspect of drug administration. Nonetheless, with the establishment of better targeting machinations comes the need for ponderings on some other important aspects, including methods to monitor NC accumulation within the system, the requirement for a toxicity standard, and even the shift of current targeted delivery towards individualized therapy. These are only some of the questions that incoming research can aim to elucidate upon.

## Data Availability

All data generated or analyzed during this study are included in this submitted version.

## Abbreviations

ABC: ATP-Binding Cassette
AIE: Aggregation-Induced Emission
Au-NP: Gold Nanoparticle
Au-NR: Gold Nanorods
BBB: Blood-Brain Barrier
CAF: Cancer-Associated Fibroblast
CNS: Central Nervous System
CNT: Carbon Nanotube
CSC: Cancer Stem Cell
CSR: Cell-Surface Receptor
CTC: Circulating Tumor Cell
DC: Dendritic Cell
DDS: Drug Delivery System
ECM: Extracellular Matrix
EGF: Epidermal Growth Factor
EPR: Enhanced Permeability and Retention
FGF: Fibroblast Growth Factor
GO: Graphene Oxide
HGF: Hepatocyte Growth Factor
HK: Hexokinase
iPSC: Induced Pluripotent Stem Cell
ICAM: Intercellular Adhesion Molecule
ICB: Immune Checkpoint Blockade
MDSC: Myeloid-Derived Suppressor Cell
MPS: Mononuclear Phagocytic System
MSC: Mesenchymal Stem Cell
NC: Nanocarrier
NEC: Neuroendocrine Cell
NP: Nanoparticle
PDGF: Platelet-Derived Growth Factor
PNP: Polymeric Nanoparticle
TAA: Tumor-Associated Antigen
TAM: Tumor-Associated Macrophages
TEC: Tumor-Associated Endothelial Cell
TGF: Transformation Growth Factor
TIL: Tumor-Infiltrating Lymphocyte
TME: Tumor Microenvironment
VEGF: Vascular Endothelial Growth Factor

## References

[1] Joyce JA, Pollard JW (2009). Microenvironmental regulation of metastasis. Nature Reviews Cancer.

[2] Balkwill FR, Capasso M, Hagemann T (2012). The tumor microenvironment at a glance. Journal of Cell Science.

[3] Arneth B (2020). Tumor microenvironment. Medicina.

[4] Henke, E., Nandigama, R., Ergün, S. (2020). Extracellular Matrix in the Tumor Microenvironment and Its Impact on Cancer Therapy. *Frontiers in Molecular Biosciences, 6*. 10.3389/fmolb.2019.00160 10.3389/fmolb.2019.00160 PMC7025524 32118030

[5] Nallanthighal S, Heiserman JP, Cheon DJ (2019). The Role of the Extracellular Matrix in Cancer Stemness. Frontiers in Cell and Developmental Biology.

[6] Islam, F., Gopalan, V., Lam, A. K. Y. (2018). Cancer stem cells: Role in tumor progression and treatment resistance. In: Oncogenomics: From Basic Research to Precision Medicine. Elsevier, pp 77–87

[7] Osman A, Afify SM, Hassan G (2020). Revisiting cancer stem cells as the origin of cancer-associated cells in the tumor microenvironment: A hypothetical view from the potential of iPSCs. Cancers (Basel).

[8] Wang M, Zhao J, Zhang L (2017). Role of tumor microenvironment in tumorigenesis. Journal of Cancer.

[9] Kalluri R (2016). The biology and function of fibroblasts in cancer. Nature Reviews Cancer.

[10] Ferguson LP, Diaz E, Reya T (2021). The Role of the Microenvironment and Immune System in Regulating Stem Cell Fate in Cancer. Trends in Cancer.

[11] Phi, L.T.H., Sari, I.N., Yang, Y.G., et al. (2018). Cancer stem cells (CSCs) in drug resistance and their therapeutic implications in cancer treatment. *Stem Cells International, 2018*. 10.1155/2018/5416923 10.1155/2018/5416923 PMC5850899 29681949

[12] Shen Q, Hill T, Cai X (2021). Physical confinement during cancer cell migration triggers therapeutic resistance and cancer stem cell-like behavior. Cancer Letters.

[13] Khan I, Saeed K, Khan I (2019). Nanoparticles: Properties, applications and toxicities. Arabian Journal of Chemistry.

[14] Fakruddin M, Hossain Z, Afroz H (2012). Prospects and applications of nanobiotechnology: A medical perspective. J Nanobiotechnology.

[15] Martins JP, das Neves J, de la Fuente M,  (2020). The solid progress of nanomedicine. Drug Delivery and Translational Research.

[16] Jena L, McErlean E, McCarthy H (2020). Delivery across the blood-brain barrier: Nanomedicine for glioblastoma multiforme. Drug Delivery and Translational Research.

[17] Valenza M, Chen JY, Di Paolo E (2015). Cholesterol-loaded nanoparticles ameliorate synaptic and cognitive function in H untington’s disease mice. EMBO Molecular Medicine.

[18] Tiwari S, Atluri V, Kaushik A (2019). Alzheimer’s disease: Pathogenesis, diagnostics, and therapeutics. International Journal of Nanomedicine.

[19] Mizrahy S, Gutkin A, Decuzzi P, Peer D (2019). Targeting central nervous system pathologies with nanomedicines. Journal of Drug Targeting.

[20] (2019) The two directions of cancer nanomedicine. *Nature Nanotechnology, 14*:1083. 10.1038/s41565-019-0597-5 10.1038/s41565-019-0597-5 31802029

[21] Germain M, Caputo F, Metcalfe S (2020). Delivering the power of nanomedicine to patients today. Journal of Controlled Release.

[22] Wilhelm S, Tavares AJ, Dai Q (2016). Analysis of nanoparticle delivery to tumours. Nature Reviews Materials.

[23] Madkour, L.H. (2019). Nanoparticle and polymeric nanoparticle-based targeted drug delivery systems. In: Nucleic Acids as Gene Anticancer Drug Delivery Therapy. Elsevier, pp 191–240

[24] Ertas, Y.N., Dorcheh, K.A., Akbari, A., Jabbari, E. (2021). Nanoparticles for targeted drug delivery to cancer stem cells: A review of recent advances. *Nanomaterials 11*. 10.3390/nano11071755 10.3390/nano11071755 PMC8308126 34361141

[25] Malachowski T, Hassel A (2020). Engineering nanoparticles to overcome immunological barriers for enhanced drug delivery. Eng Regen.

[26] Valdivia-Olivares, R.Y., Rodriguez-Fernandez, M., Álvarez-Figueroa, M.J., et al. (2021). The importance of nanocarrier design and composition for an efficient nanoparticle-mediated transdermal vaccination. *Vaccines 9*. 10.3390/vaccines9121420 10.3390/vaccines9121420 PMC8705631 34960166

[27] Navya PN, Kaphle A, Srinivas SP (2019). Current trends and challenges in cancer management and therapy using designer nanomaterials. Nano Convergence.

[28] Blanco E, Hsiao A, Ruiz-Esparza GU (2011). Molecular-targeted nanotherapies in cancer: Enabling treatment specificity. Molecular Oncology.

[29] Hsu FT, Wei ZH, Hsuan YCY (2018). MRI tracking of polyethylene glycol-coated superparamagnetic iron oxide-labelled placenta-derived mesenchymal stem cells toward glioblastoma stem-like cells in a mouse model. Artif Cells, Nanomedicine Biotechnol.

[30] Guldris N, Argibay B, Gallo J (2017). Magnetite Nanoparticles for Stem Cell Labeling with High Efficiency and Long-Term in Vivo Tracking. Bioconjugate Chemistry.

[31] Azevedo-Pereira RL, Rangel B, Tovar-Moll F (2019). Superparamagnetic iron oxide nanoparticles as a tool to track mouse neural stem cells in vivo. Molecular Biology Reports.

[32] Li K, Qin W, Ding D (2013). Photostable fluorescent organic dots with aggregation-induced emission (AIE dots) for noninvasive long-term cell tracing. Science and Reports.

[33] Sung H, Ferlay J, Siegel RL (2021). Global Cancer Statistics 2020: GLOBOCAN Estimates of Incidence and Mortality Worldwide for 36 Cancers in 185 Countries. CA: A Cancer Journal for Clinicians.

[34] Cacho-Díaz B, García-Botello DR, Wegman-Ostrosky T (2020). Tumor microenvironment differences between primary tumor and brain metastases. Journal of Translational Medicine.

[35] Zhang R, Liu Q, Li T (2019). Role of the complement system in the tumor microenvironment. Cancer Cell International.

[36] Lambert AW, Pattabiraman DR, Weinberg RA (2017). Emerging Biological Principles of Metastasis. Cell.

[37] Pietilä M, Ivaska J, Mani SA (2016). Whom to blame for metastasis, the epithelial–mesenchymal transition or the tumor microenvironment?. Cancer Letters.

[38] Catalán, V., Gómez-Ambrosi, J., Rodríguez, A., Frühbeck, G. (2013). Adipose tissue immunity and cancer. *Frontiers in Physiology 4 *OCT. 10.3389/fphys.2013.00275 10.3389/fphys.2013.00275 PMC3788329 24106481

[39] Cozzo AJ, Fuller AM, Makowski L (2018). Contribution of adipose tissue to development of cancer. Comprehensive Physiology.

[40] LeBleu, V.S., Kalluri, R. (2018). A peek into cancer-associated fibroblasts: Origins, functions and translational impact. *Disease Models & Mechanisms, 11*. 10.1242/dmm.029447 10.1242/dmm.029447 PMC5963854 29686035

[41] Liu T, Zhou L, Li D (2019). Cancer-associated fibroblasts build and secure the tumor microenvironment. Frontiers in Cell and Developmental Biology.

[42] Albini A, Bruno A, Noonan DM, Mortara L (2018). Contribution to tumor angiogenesis from innate immune cells within the tumor microenvironment: Implications for immunotherapy. Frontiers in Immunology.

[43] Hanahan D, Coussens LM (2012). Accessories to the Crime: Functions of Cells Recruited to the Tumor Microenvironment. Cancer Cell.

[44] Picoli CC, Gonçalves BÔP, Santos GSP (2021). Pericytes cross-talks within the tumor microenvironment. Biochimica et Biophysica Acta - Reviews on Cancer.

[45] Ribatti D, Tamma R, Annese T (2020). Epithelial-Mesenchymal Transition in Cancer: A Historical Overview. Translational Oncology.

[46] Szekely B, Bossuyt V, Li X (2018). Immunological differences between primary and metastatic breast cancer. Annals of Oncology.

[47] Zeppellini A, Galimberti S, Leone BE (2021). Comparison of tumor microenvironment in primary and paired metastatic ER+/HER2- breast cancers: Results of a pilot study. BMC Cancer.

[48] Heeke S, Mograbi B, Alix-Panabières C, Hofman P (2019). Never Travel Alone: The Crosstalk of Circulating Tumor Cells and the Blood Microenvironment. Cells.

[49] Leone K, Poggiana C, Zamarchi R (2018). The Interplay between Circulating Tumor Cells and the Immune System: From Immune Escape to Cancer Immunotherapy. Diagnostics.

[50] Wang J, Li D, Cang H, Guo B (2019). Crosstalk between cancer and immune cells: Role of tumor-associated macrophages in the tumor microenvironment. Cancer Medicine.

[51] Rahat, M.A., Shakya, J. (2016). Parallel Aspects of the Microenvironment in Cancer and Autoimmune Disease. *Mediators Inflamm, 2016*. 10.1155/2016/4375120 10.1155/2016/4375120 PMC4779817 26997761

[52] Pearce OMT, Delaine-Smith RM, Maniati E (2018). Deconstruction of a metastatic tumor microenvironment reveals a common matrix response in human cancers. Cancer Discovery.

[53] Labani-Motlagh A, Ashja-Mahdavi M, Loskog A (2020). The Tumor Microenvironment: A Milieu Hindering and Obstructing Antitumor Immune Responses. Frontiers in Immunology.

[54] Idos GE, Kwok J, Bonthala N (2020). The Prognostic Implications of Tumor Infiltrating Lymphocytes in Colorectal Cancer: A Systematic Review and Meta-Analysis. Science and Reports.

[55] Quail DF, Joyce JA (2013). Microenvironmental regulation of tumor progression and metastasis. Nature Medicine.

[56] Ajani JA, Song S, Hochster HS, Steinberg IB (2015). Cancer stem cells: The promise and the potential. Seminars in Oncology.

[57] Najafi M, Farhood B, Mortezaee K (2019). Cancer stem cells (CSCs) in cancer progression and therapy. Journal of Cellular Physiology.

[58] Yang L, Shi P, Zhao G (2020). Targeting cancer stem cell pathways for cancer therapy. Signal Transduction and Targeted Therapy.

[59] Afify SM, Seno M (2019). Conversion of stem cells to cancer stem cells: Undercurrent of cancer initiation. Cancers (Basel).

[60] Zhu P, Fan Z (2018). Cancer stem cells and tumorigenesis. Biophys Reports.

[61] Gasch C, Ffrench B, O’Leary JJ (2017). Gallagher MF (2017) Catching moving targets: Cancer stem cell hierarchies, therapy-resistance & considerations for clinical intervention. Molecular Cancer.

[62] Kreso A, Dick JE (2014). Evolution of the Cancer Stem Cell Model. Cell Stem Cell.

[63] Zheng X, Yu C, Xu M (2021). Linking Tumor Microenvironment to Plasticity of Cancer Stem Cells: Mechanisms and Application in Cancer Therapy. Frontiers in Oncology.

[64] Hikichi T, Matoba R, Ikeda T (2013). Transcription factors interfering with dedifferentiation induce cell type-specific transcriptional profiles. Proc Natl Acad Sci U S A.

[65] Poli, V., Fagnocchi, L., Zippo, A. (2018). Tumorigenic Cell Reprogramming and Cancer Plasticity: Interplay between Signaling, Microenvironment, and Epigenetics. *Stem Cells International, 2018*. 10.1155/2018/4598195 10.1155/2018/4598195 PMC5954911 29853913

[66] French R, Pauklin S (2021). Epigenetic regulation of cancer stem cell formation and maintenance. International Journal of Cancer.

[67] Walcher L, Kistenmacher AK, Suo H (2020). Cancer Stem Cells—Origins and Biomarkers: Perspectives for Targeted Personalized Therapies. Frontiers in Immunology.

[68] Kaushik V, Kulkarni Y, Felix K (2021). Alternative models of cancer stem cells: The stemness phenotype model, 10 years later. World J Stem Cells.

[69] Nair N, Calle AS, Zahra MH (2017). A cancer stem cell model as the point of origin of cancer-associated fibroblasts in tumor microenvironment. Science and Reports.

[70] Sadozai, H., Acharjee, A., Eppenberger-Castori, S., et al. (2021). Distinct Stromal and Immune Features Collectively Contribute to Long-Term Survival in Pancreatic Cancer. *Frontiers in Immunology, 12*. 10.3389/fimmu.2021.643529 10.3389/fimmu.2021.643529 PMC7933000 33679807

[71] Song M, He J, Pan QZ (2021). Cancer-Associated Fibroblast-Mediated Cellular Crosstalk Supports Hepatocellular Carcinoma Progression. Hepatology.

[72] Wei, L., Ye, H., Li, G., et al. (2018). Cancer-associated fibroblasts promote progression and gemcitabine resistance via the SDF-1/SATB-1 pathway in pancreatic cancer. *Cell Death & Disease, 9*. 10.1038/s41419-018-1104-x 10.1038/s41419-018-1104-x PMC6194073 30337520

[73] Das PK, Pillai S, Rakib MA (2020). Plasticity of Cancer Stem Cell: Origin and Role in Disease Progression and Therapy Resistance. Stem Cell Rev Reports.

[74] Cazet, A.S., Hui, M.N., Elsworth, B.L., et al. (2018). Targeting stromal remodeling and cancer stem cell plasticity overcomes chemoresistance in triple negative breast cancer. *Nature Communications, 9*. 10.1038/s41467-018-05220-6 10.1038/s41467-018-05220-6 PMC6057940 30042390

[75] Strell C, Stenmark Tullberg A, Jetne Edelmann R (2021). Prognostic and predictive impact of stroma cells defined by PDGFRb expression in early breast cancer: Results from the randomized SweBCG91RT trial. Breast Cancer Research and Treatment.

[76] Codd AS, Kanaseki T, Torigo T, Tabi Z (2018). Cancer stem cells as targets for immunotherapy. Immunology.

[77] Cho Y, Kim YK (2020). Cancer Stem Cells as a Potential Target to Overcome Multidrug Resistance. Frontiers in Oncology.

[78] Zhou HM, Zhang JG, Zhang X (2021). Li Q (2021) Targeting cancer stem cells for reversing therapy resistance: Mechanism, signaling, and prospective agents. Signal Transduction and Targeted Therapy.

[79] Merchant AA, Matsui W (2010). Targeting Hedgehog - A cancer stem cell pathway. Clinical Cancer Research.

[80] Gulino A, Ferretti E, De Smaele E (2009). Hedgehog signalling in colon cancer and stem cells. EMBO Molecular Medicine.

[81] Jia Y, Wang Y, Xie J (2015). The Hedgehog pathway: Role in cell differentiation, polarity and proliferation. Archives of Toxicology.

[82] Wu C, Zhu X, Liu W (2017). Hedgehog signaling pathway in colorectal cancer: Function, mechanism, and therapy. Oncotargets and Therapy.

[83] Safa AR (2016). Resistance to cell death and its modulation in cancer stem cells. Critical Reviews in Oncogenesis.

[84] BeLow M, Osipo C (2020). Notch Signaling in Breast Cancer: A Role in Drug Resistance. Cells.

[85] Lin X, Sun B, Zhu D (2016). Notch4+ cancer stem-like cells promote the metastatic and invasive ability of melanoma. Cancer Science.

[86] Rajendran DT, Subramaniyan B, Ganeshan M (2018) Role of notch signaling in colorectal cancer. In: Role of Transcription Factors in Gastrointestinal Malignancies. pp 305–312

[87] Meisel CT, Porcheri C, Mitsiadis TA (2020). Cancer Stem Cells, Quo Vadis? The Notch Signaling Pathway in Tumor Initiation and Progression. Cells.

[88] Nami B, Wang Z (2017). HER2 in breast cancer stemness: A negative feedback loop towards trastuzumab resistance. Cancers (Basel).

[89] Duchartre Y, Kim YM, Kahn M (2016). The Wnt signaling pathway in cancer. Critical Reviews in Oncology Hematology.

[90] Yan, Y., Liu, F., Han, L., et al. (2018). HIF-2α promotes conversion to a stem cell phenotype and induces chemoresistance in breast cancer cells by activating Wnt and Notch pathways. *Journal of Experimental & Clinical Cancer Research, 37*. 10.1186/s13046-018-0925-x 10.1186/s13046-018-0925-x PMC6194720 30340507

[91] Nagata T, Shimada Y, Sekine S (2017). KLF4 and NANOG are prognostic biomarkers for triple-negative breast cancer. Breast Cancer.

[92] Najafzadeh B, Asadzadeh Z, Motafakker Azad R (2021). The oncogenic potential of NANOG: An important cancer induction mediator. Journal of Cellular Physiology.

[93] House CD, Jordan E, Hernandez L (2017). NFkB promotes ovarian tumorigenesis via classical pathways that support proliferative cancer cells and alternative pathways that support ALDHþ cancer stem–like cells. Cancer Research.

[94] Volmar M, Cheng J, Synowitz M (2021). OMRT-1. Cannabidiol converts NFKB into a tumor-suppressor in glioblastoma with defined antioxidative properties. Neuro-Oncology Advances.

[95] Gimple RC, Wang X (2019). RAS: Striking at the Core of the Oncogenic Circuitry. Frontiers in Oncology.

[96] Chippalkatti R, Abankwa D (2021). Promotion of cancer cell stemness by Ras. Biochemical Society Transactions.

[97] Mulholland DJ, Kobayashi N, Ruscetti M (2012). Pten loss and RAS/MAPK activation cooperate to promote EMT and metastasis initiated from prostate cancer stem/progenitor cells. Cancer Research.

[98] Vitale G, Zappavigna S, Marra M (2012). The PPAR-γ agonist troglitazone antagonizes survival pathways induced by STAT-3 in recombinant interferon-β treated pancreatic cancer cells. Biotechnology Advances.

[99] Xu M, Wang S, Wang Y (2018). Role of p38γ MAPK in Regulation of EMT and Cancer Stem Cells. Biochimica et Biophysica Acta, Molecular Basis of Disease.

[100] Li J, Wang J, Xie D (2021). Characteristics of the PI3K/AKT and MAPK/ERK pathways involved in the maintenance of self-renewal in lung cancer stem-like cells. International Journal of Biological Sciences.

[101] Velázquez-Quesada I, Ruiz-Moreno AJ, Casique-Aguirre D (2020). Pranlukast antagonizes cd49f and reduces stemness in triple-negative breast cancer cells. Drug Design, Development and Therapy.

[102] Madsen RR (2020). PI3K in stemness regulation: From development to cancer. Biochemical Society Transactions.

[103] Liffers, K., Lamszus, K., Schulte, A. (2015). EGFR Amplification and Glioblastoma Stem-Like Cells. *Stem Cells International, 2015*. 10.1155/2015/427518 10.1155/2015/427518 PMC4468289 26136784

[104] Wang Y, Han Y, Xu S (2020). Targeting EGFR Enriches Stem Cell-Like Properties in Salivary Adenoid Cystic Carcinoma by Activating the Notch1 Pathway</p>. Cancer Manag Res.

[105] Xu Y, Afify SM, Du J (2022). (2022) The efficacy of PI3Kγ and EGFR inhibitors on the suppression of the characteristics of cancer stem cells. Sci Reports.

[106] Ayob AZ (2018). Ramasamy TS (2018) Cancer stem cells as key drivers of tumour progression. Journal of Biomedical Science.

[107] Aramini, B., Masciale, V., Grisendi, G., et al. (2022). Dissecting Tumor Growth: The Role of Cancer Stem Cells in Drug Resistance and Recurrence. *Cancers (Basel), 14*. 10.3390/CANCERS14040976 10.3390/cancers14040976 PMC8869911 35205721

[108] Kahn, B.M., Lucas, A., Alur, R.G., et al. (2021). The vascular landscape of human cancer. *The Journal of Clinical Investigation*. 10.1172/JCI136655 10.1172/JCI136655 PMC7810485 33258803

[109] Lugano R, Ramachandran M, Dimberg A (2020). Tumor angiogenesis: Causes, consequences, challenges and opportunities. Cellular and Molecular Life Sciences.

[110] Tsuchiya H, Shiota G (2021). Immune evasion by cancer stem cells. Regenerative Therapy.

[111] Chen J, Chen S, Zhuo L (2020). (2020) Regulation of cancer stem cell properties, angiogenesis, and vasculogenic mimicry by miR-450a-5p/SOX2 axis in colorectal cancer. Cell Death & Disease.

[112] Baruah J, Wary KK (2020). Exosomes in the Regulation of Vascular Endothelial Cell Regeneration. Front Cell Dev Biol.

[113] Ahmadi M, Rezaie J (2020). Tumor cells derived-exosomes as angiogenenic agents: Possible therapeutic implications. Journal of Translational Medicine.

[114] Mongiat, M., Andreuzzi, E., Tarticchio, G., Paulitti, A. (2016). Extracellular Matrix, a Hard Player in Angiogenesis. *International Journal of Molecular Sciences, 17*. 10.3390/IJMS17111822 10.3390/ijms17111822 PMC5133823 27809279

[115] Radomska-Leśniewska, D. M., Białoszewska, A., Kamiński, P. (2021). Angiogenic Properties of NK Cells in Cancer and Other Angiogenesis-Dependent Diseases. *Cells ,10*. 10.3390/CELLS10071621 10.3390/cells10071621 PMC8303392 34209508

[116] Fares J, Fares MY, Khachfe HH (2020). (2020) Molecular principles of metastasis: A hallmark of cancer revisited. Signal Transduction and Targeted Therapy.

[117] Sun X, Ma X, Wang J (2017). Glioma stem cells-derived exosomes promote the angiogenic ability of endothelial cells through miR-21/VEGF signal. Oncotarget.

[118] Treps, L., Perret, R., Edmond, S., et al. (2017). Glioblastoma stem-like cells secrete the pro-angiogenic VEGF-A factor in extracellular vesicles. *Journal of Extracellular Vesicles, 6*. 10.1080/20013078.2017.1359479 10.1080/20013078.2017.1359479 PMC5549846 28815003

[119] Lizárraga-Verdugo E, Avendaño-Félix M, Bermúdez M (2020). Cancer Stem Cells and Its Role in Angiogenesis and Vasculogenic Mimicry in Gastrointestinal Cancers. Frontiers in Oncology.

[120] Randi AM, Smith KE, Castaman G (2018). von Willebrand factor regulation of blood vessel formation. Blood.

[121] Patmore S, Dhami SPS, O’Sullivan JM (2020). Von Willebrand factor and cancer; metastasis and coagulopathies. Journal of Thrombosis and Haemostasis.

[122] Goh CY, Patmore S, Smolenski A (2021). The role of von Willebrand factor in breast cancer metastasis. Transl Oncol.

[123] Li X, Lu Z (2022). Role of von Willebrand factor in the angiogenesis of lung adenocarcinoma (Review). Oncology Letters.

[124] Wang T, Wang X, Wang H (2021). High TSPAN8 expression in epithelial cancer cell-derived small extracellular vesicles promote confined diffusion and pronounced uptake. J Extracell Vesicles.

[125] Mu, W., Provaznik, J., Hackert, T., Zöller, M. (2020). Tspan8-Tumor Extracellular Vesicle-Induced Endothelial Cell and Fibroblast Remodeling Relies on the Target Cell-Selective Response. *Cells, 9*. 10.3390/CELLS9020319 10.3390/cells9020319 PMC7072212 32013145

[126] Chen C, Xu ZQ, Zong YP (2019). CXCL5 induces tumor angiogenesis via enhancing the expression of FOXD1 mediated by the AKT/NF-κB pathway in colorectal cancer. Cell Death & Disease.

[127] Zhang W, Wang H, Sun M (2020). CXCL5/CXCR2 axis in tumor microenvironment as potential diagnostic biomarker and therapeutic target. Cancer Communications.

[128] Nie Y, Jiang MC, Liu C (2021). CXCL5 Has Potential to Be a Marker for Hepatocellular Carcinoma Prognosis and Was Correlating With Immune Infiltrates. Frontiers in Oncology.

[129] Macías M, García-Cortés Á, Torres M (2021). Characterization of the perioperative changes of exosomal immune-related cytokines induced by prostatectomy in early-stage prostate cancer patients. Cytokine.

[130] Soumoy L, Kindt N, Ghanem G (2019). Role of Macrophage Migration Inhibitory Factor (MIF) in Melanoma. Cancers.

[131] Noe JT, Mitchell RA (2020). MIF-Dependent Control of Tumor Immunity. Frontiers in Immunology.

[132] Klemke L, De Oliveira T, Witt D (2021). (2021) Hsp90-stabilized MIF supports tumor progression via macrophage recruitment and angiogenesis in colorectal cancer. Cell Death & Disease.

[133] Ambrosini G, Rai AJ, Carvajal RD, Schwartz GK (2022). Uveal Melanoma Exosomes Induce a Prometastatic Microenvironment through Macrophage Migration Inhibitory Factor. Molecular Cancer Research.

[134] Korbecki J, Kojder K, Simińska D (2020). CC Chemokines in a Tumor: A Review of Pro-Cancer and Anti-Cancer Properties of the Ligands of Receptors CCR1, CCR2, CCR3, and CCR4. International Journal of Molecular Sciences.

[135] Bule, P., Aguiar, S. I., Aires-Da-silva, F., Dias, J. N. R. (2021). Chemokine-Directed Tumor Microenvironment Modulation in Cancer Immunotherapy. *International Journal of Molecular Sciences, 22*. 10.3390/IJMS22189804 10.3390/ijms22189804 PMC8464715 34575965

[136] Gilchrist A, Echeverria SL (2022). Targeting Chemokine Receptor CCR1 as a Potential Therapeutic Approach for Multiple Myeloma. Front Endocrinol (Lausanne).

[137] Liu J, Ren L, Li S (2021). The biology, function, and applications of exosomes in cancer. Acta Pharm Sin B.

[138] Ruehle, M. A., Eastburn, E. A., LaBelle, S. A., et al. (2020). Extracellular matrix compression temporally regulates microvascular angiogenesis. *Science Advances, 6*. 10.1126/SCIADV.ABB6351/SUPPL_FILE/ABB6351_SM.PDF 10.1126/sciadv.abb6351 PMC7442478 32937368

[139] Abou Khouzam R, Brodaczewska K, Filipiak A (2021). Tumor Hypoxia Regulates Immune Escape/Invasion: Influence on Angiogenesis and Potential Impact of Hypoxic Biomarkers on Cancer Therapies. Frontiers in Immunology.

[140] Kim JH, Verwilst P, Won M (2021). A Small Molecule Strategy for Targeting Cancer Stem Cells in Hypoxic Microenvironments and Preventing Tumorigenesis. Journal of the American Chemical Society.

[141] Bhuria, V., Xing, J., Scholta, T., et al. (2019). Hypoxia induced Sonic Hedgehog signaling regulates cancer stemness, epithelial-to-mesenchymal transition and invasion in cholangiocarcinoma. *Experimental Cell Research, 385*. 10.1016/J.YEXCR.2019.111671 10.1016/j.yexcr.2019.111671 31634481

[142] Nascimento-Filho CHV, Webber LP, Borgato GB (2019). Hypoxic niches are endowed with a protumorigenic mechanism that supersedes the protective function of PTEN. The FASEB Journal.

[143] Zhang, Q., Han, Z., Zhu, Y., et al. (2021). Role of hypoxia inducible factor-1 in cancer stem cells. *Molecular Medicine Reports, 23*. 10.3892/MMR.2020.11655 10.3892/mmr.2020.11655 PMC7673349 33179080

[144] Huang, Y., Chen, Z., Lu, T., et al. (2021). HIF-1α switches the functionality of TGF-β signaling via changing the partners of smads to drive glucose metabolic reprogramming in non-small cell lung cancer. *Journal of Experimental & Clinical Cancer Research, 40*. 10.1186/S13046-021-02188-Y 10.1186/s13046-021-02188-y PMC8690885 34930376

[145] Tam, S.Y., Wu, V. W. C., Law, H. K. W. (2020). Hypoxia-Induced Epithelial-Mesenchymal Transition in Cancers: HIF-1α and Beyond. *Frontiers in Oncology, 10*. 10.3389/FONC.2020.00486 10.3389/fonc.2020.00486 PMC7156534 32322559

[146] Peng J, Wang X, Ran L (2018). Hypoxia-Inducible Factor 1α Regulates the Transforming Growth Factor β1/SMAD Family Member 3 Pathway to Promote Breast Cancer Progression. Journal of Breast Cancer.

[147] Zhang Q, Bai X, Chen W (2013). Wnt/β-catenin signaling enhances hypoxia-induced epithelial-mesenchymal transition in hepatocellular carcinoma via crosstalk with hif-1α signaling. Carcinogenesis.

[148] Lin, Y. Te, Wu, K. J. (2020). Epigenetic regulation of epithelial-mesenchymal transition: focusing on hypoxia and TGF-β signaling. *Journal of Biomedical Science, 27*. 10.1186/S12929-020-00632-3 10.1186/s12929-020-00632-3 PMC7050137 32114978

[149] Fu, Y., Bao, Q., Liu, Z., et al. (2021). Development and Validation of a Hypoxia-Associated Prognostic Signature Related to Osteosarcoma Metastasis and Immune Infiltration. *Frontiers in Cell and Developmental Biology, 9*. 10.3389/FCELL.2021.633607 10.3389/fcell.2021.633607 PMC8012854 33816483

[150] Pezzuto A, Carico E (2018). Role of HIF-1 in Cancer Progression: Novel Insights. A Review. Curr Mol Med.

[151] Sun H, Meng Q, Shi C (2021). Hypoxia-Inducible Exosomes Facilitate Liver-Tropic Premetastatic Niche in Colorectal Cancer. Hepatology.

[152] Vander Linden, C., Corbet, C. (2019). Therapeutic Targeting of Cancer Stem Cells: Integrating and Exploiting the Acidic Niche. *Frontiers in Oncology, 9*. 10.3389/FONC.2019.00159 10.3389/fonc.2019.00159 PMC6433943 30941310

[153] Emami Nejad A, Najafgholian S, Rostami A (2021). (2021) The role of hypoxia in the tumor microenvironment and development of cancer stem cell: A novel approach to developing treatment. Cancer Cell International.

[154] Lv X, Li J, Zhang C (2017). The role of hypoxia-inducible factors in tumor angiogenesis and cell metabolism. Genes Dis.

[155] Wei X, Chen Y, Jiang X (2021). (2021) Mechanisms of vasculogenic mimicry in hypoxic tumor microenvironments. Molecular Cancer.

[156] Gong, P. J., Shao, Y. C., Huang, S. R., et al. (2020). Hypoxia-Associated Prognostic Markers and Competing Endogenous RNA Co-Expression Networks in Breast Cancer. *Frontiers in Oncology, 10*. 10.3389/FONC.2020.579868 10.3389/fonc.2020.579868 PMC7738636 33344235

[157] Liu Z, Wang Y, Dou C (2018). Hypoxia-induced up-regulation of VASP promotes invasiveness and metastasis of hepatocellular carcinoma. Theranostics.

[158] Yoshimoto S, Tanaka F, Morita H (2019). Hypoxia-induced HIF-1α and ZEB1 are critical for the malignant transformation of ameloblastoma via TGF-β-dependent EMT. Cancer Medicine.

[159] Su, Q., Fan, M., Wang, J., et al. (2019). Sanguinarine inhibits epithelial-mesenchymal transition via targeting HIF-1α/TGF-β feed-forward loop in hepatocellular carcinoma. *Cell Death & Disease, 10*. 10.1038/S41419-019-2173-1 10.1038/s41419-019-2173-1 PMC6901539 31819036

[160] Xiong X, Sun Y, Wang X (2020). HIF1A/miR-20a-5p/TGFβ1 axis modulates adipose-derived stem cells in a paracrine manner to affect the angiogenesis of human dermal microvascular endothelial cells. Journal of Cellular Physiology.

[161] Pang L, Tian P, Cui X (2021). In Situ Photo-Cross-Linking Hydrogel Accelerates Diabetic Wound Healing through Restored Hypoxia-Inducible Factor 1-Alpha Pathway and Regulated Inflammation. ACS Applied Materials & Interfaces.

[162] De Francesco, E. M., Maggiolini, M., Musti, A. M. (2018). Crosstalk between Notch, HIF-1α and GPER in Breast Cancer EMT. *International Journal of Molecular Sciences, 19*. 10.3390/IJMS19072011 10.3390/ijms19072011 PMC6073901 29996493

[163] Zhang HS, Zhang ZG, Du GY (2019). Nrf2 promotes breast cancer cell migration via up-regulation of G6PD/HIF-1α/Notch1 axis. Journal of Cellular and Molecular Medicine.

[164] Liu ZZ, Tian YF, Wu H (2020). LncRNA H19 promotes glioma angiogenesis through miR-138/HIF-1α/VEGF axis. Neoplasma.

[165] Huang YH, Kuo CH, Peng IC (2021). Recombinant thrombomodulin domain 1 rescues pathological angiogenesis by inhibition of HIF-1α-VEGF pathway. Cellular and Molecular Life Sciences.

[166] Xu, Z., Zhu, C., Chen, C., et al. (2018). CCL19 suppresses angiogenesis through promoting miR-206 and inhibiting Met/ERK/Elk-1/HIF-1α/VEGF-A pathway in colorectal cancer. *Cell Death & Disease, 9*. 10.1038/S41419-018-1010-2 10.1038/s41419-018-1010-2 PMC6155262 30250188

[167] Hong, J., Kim, Y., Yanpallewar, S., Charles Lin, P. (2020). The Rho/Rac Guanine Nucleotide Exchange Factor Vav1 Regulates Hif-1α and Glut-1 Expression and Glucose Uptake in the Brain. *International Journal of Molecular Sciences, 21*. 10.3390/IJMS21041341 10.3390/ijms21041341 PMC7072975 32079227

[168] Tang W, Long T, Li F (2021). HIF - 1 α may promote glycolysis in psoriasis vulgaris via upregulation of CD147 and GLUT1. Zhong Nan Da Xue Xue Bao Yi Xue Ban.

[169] Al Tameemi W, Dale TP, Al-Jumaily RMK, Forsyth NR (2019). Hypoxia-Modified Cancer Cell Metabolism. Frontiers in Cell and Developmental Biology.

[170] Carrasco-Pozo, C., Tan, K. N., Rodriguez, T., Avery, V. M. (2019). The Molecular Effects of Sulforaphane and Capsaicin on Metabolism upon Androgen and Tip60 Activation of Androgen Receptor. *International Journal of Molecular Sciences, 20*. 10.3390/IJMS20215384 10.3390/ijms20215384 PMC6861939 31671779

[171] Ikeda S, Abe F, Matsuda Y (2020). Hypoxia-inducible hexokinase-2 enhances anti-apoptotic function via activating autophagy in multiple myeloma. Cancer Science.

[172] Du D, Liu C, Qin M (2022). Metabolic dysregulation and emerging therapeutical targets for hepatocellular carcinoma. Acta Pharmaceutica Sinica B.

[173] Tse, A. P. W., Sze, K. M. F., Shea, Q. T. K., et al. (2018). Hepatitis transactivator protein X promotes extracellular matrix modification through HIF/LOX pathway in liver cancer. *Oncogenesis, 7*. 10.1038/S41389-018-0052-8 10.1038/s41389-018-0052-8 PMC5968027 29799025

[174] Li, Z., Shi, L., Li, X., et al. (2021). RNF144A-AS1, a TGF-β1- and hypoxia-inducible gene that promotes tumor metastasis and proliferation via targeting the miR-30c-2-3p/LOX axis in gastric cancer. *Cell & Bioscience, 11*. 10.1186/S13578-021-00689-Z 10.1186/s13578-021-00689-z PMC8480077 34583752

[175] Murdocca M, De Masi C, Pucci S (2021). LOX-1 and cancer: An indissoluble liaison. Cancer Gene Therapy.

[176] Yeo CD, Kang N, Choi SY (2017). The role of hypoxia on the acquisition of epithelial-mesenchymal transition and cancer stemness: A possible link to epigenetic regulation. Korean Journal of Internal Medicine.

[177] Dong, W., Kong, M., Zhu, Y., et al. (2020). Activation of TWIST Transcription by Chromatin Remodeling Protein BRG1 Contributes to Liver Fibrosis in Mice. *Frontiers in Cell and Developmental Biology, 8*. 10.3389/FCELL.2020.00340 10.3389/fcell.2020.00340 PMC7237740 32478075

[178] Wang, Q., He, Z., Huang, M., et al. (2018). Vascular niche IL-6 induces alternative macrophage activation in glioblastoma through HIF-2α. *Nature Communications, 9*. 10.1038/S41467-018-03050-0 10.1038/s41467-018-03050-0 PMC5805734 29422647

[179] Xu K, Zhan Y, Yuan Z (2019). Hypoxia Induces Drug Resistance in Colorectal Cancer through the HIF-1α/miR-338-5p/IL-6 Feedback Loop. Molecular Therapy.

[180] Su Q, Wang J, Fan M (2020). Sanguinarine disrupts the colocalization and interaction of HIF-1α with tyrosine and serine phosphorylated-STAT3 in breast cancer. Journal of Cellular and Molecular Medicine.

[181] Zhang J, Fan J, Zeng X (2021). Hedgehog signaling in gastrointestinal carcinogenesis and the gastrointestinal tumor microenvironment. Acta Pharmaceutica Sinica B.

[182] Yang, X., Zheng, Y., Tan, J., et al. (2021). MiR-199a-5p-HIF-1α-STAT3 Positive Feedback Loop Contributes to the Progression of Non-Small Cell Lung Cancer. *Frontiers in Cell and Developmental Biology, 8*. 10.3389/FCELL.2020.620615 10.3389/fcell.2020.620615 PMC7929999 33681184

[183] Cao, J., Li, L., Xiong, L., et al. (2022). Research on the mechanism of berberine in the treatment of COVID-19 pneumonia pulmonary fibrosis using network pharmacology and molecular docking. *Phytomedicine Plus : International Journal of Phytotherapy and Phytopharmacology, 2*. 10.1016/J.PHYPLU.2022.100252 10.1016/j.phyplu.2022.100252 PMC8895682 35403089

[184] Liang Z, Chi YJ, Lin GQ (2018). MiRNA-26a promotes angiogenesis in a rat model of cerebral infarction via PI3K/AKT and MAPK/ERK pathway. European Review for Medical and Pharmacological Sciences.

[185] Xu, X., You, K., Bu, R. (2019). Proximal Tubular Development Is Impaired with Downregulation of MAPK/ERK Signaling, HIF-1 α, and Catalase by Hyperoxia Exposure in Neonatal Rats. *Oxidative Medicine and Cellular Longevity, 2019*. 10.1155/2019/9219847 10.1155/2019/9219847 PMC6735195 31558952

[186] Russignan, A., Dal Collo, G., Bagnato, A., et al. (2021). Targeting the Endothelin-1 Receptors Curtails Tumor Growth and Angiogenesis in Multiple Myeloma. *Frontiers in Oncology, 10*. 10.3389/FONC.2020.600025 10.3389/fonc.2020.600025 PMC7820698 33489901

[187] Wang, P., Zhao, L., Gong, S., et al. (2021). HIF1α/HIF2α-Sox2/Klf4 promotes the malignant progression of glioblastoma via the EGFR-PI3K/AKT signalling pathway with positive feedback under hypoxia. *Cell Death & Disease 12*. 10.1038/S41419-021-03598-8 10.1038/s41419-021-03598-8 PMC7990922 33762574

[188] Wang, Y., Bibi, M., Min, P., et al. (2019). SOX2 promotes hypoxia-induced breast cancer cell migration by inducing NEDD9 expression and subsequent activation of Rac1/HIF-1α signaling. *Cellular & Molecular Biology Letters, 24*. 10.1186/S11658-019-0180-Y 10.1186/s11658-019-0180-y PMC6704701 31462898

[189] Chen, G., Liu, B., Yin, S., et al. (2020). Hypoxia induces an endometrial cancer stem-like cell phenotype via HIF-dependent demethylation of SOX2 mRNA. *Oncogenesis, 9*. 10.1038/S41389-020-00265-Z 10.1038/s41389-020-00265-z PMC7484801 32913192

[190] Li, Q., Sun, H., Luo, D., et al. (2021). Lnc-RP11–536 K7.3/SOX2/HIF-1α signaling axis regulates oxaliplatin resistance in patient-derived colorectal cancer organoids. *Journal of Experimental & Clinical Cancer Research, 40*. 10.1186/S13046-021-02143-X 10.1186/s13046-021-02143-x PMC8570024 34740372

[191] Kuo YC, Au HK, Hsu JL (2018). IGF-1R Promotes Symmetric Self-Renewal and Migration of Alkaline Phosphatase + Germ Stem Cells through HIF-2α-OCT4/CXCR4 Loop under Hypoxia. Stem Cell Reports.

[192] Jiang Z, Zhang C, Liu X (2020). Dexamethasone inhibits stemness maintenance and enhances chemosensitivity of hepatocellular carcinoma stem cells by inducing deSUMOylation of HIF-1α and Oct4. International Journal of Oncology.

[193] Lu H, Xie Y, Tran L (2020). Chemotherapy-induced S100A10 recruits KDM6A to facilitate OCT4-mediated breast cancer stemness. The Journal of Clinical Investigation.

[194] Jiang Y, Mao C, Yang R (2017). EGLN1/c-Myc Induced Lymphoid-Specific Helicase Inhibits Ferroptosis through Lipid Metabolic Gene Expression Changes. Theranostics.

[195] Boldrini L, Bartoletti R, Giordano M (2019). C-MYC, HIF-1α, ERG, TKT, and GSTP1: An Axis in Prostate Cancer?. Pathology Oncology Research.

[196] Liu, X., Zhou, Y., Peng, J., et al. (2020). Silencing c-Myc Enhances the Antitumor Activity of Bufalin by Suppressing the HIF-1α/SDF-1/CXCR4 Pathway in Pancreatic Cancer Cells. *Frontiers in Pharmacology, 11*. 10.3389/FPHAR.2020.00495 10.3389/fphar.2020.00495 PMC7181899 32362830

[197] Li, Y., Sun, X. X., Qian, D. Z., Dai, M. S. (2020). Molecular Crosstalk Between MYC and HIF in Cancer. *Frontiers in Cell and Developmental Biology, 8*. 10.3389/FCELL.2020.590576 10.3389/fcell.2020.590576 PMC7676913 33251216

[198] Mao Y, Wang Y, Dong L (2019). Hypoxic exosomes facilitate angiogenesis and metastasis in esophageal squamous cell carcinoma through altering the phenotype and transcriptome of endothelial cells. Journal of Experimental & Clinical Cancer Research.

[199] Li, C., Teixeira, A. F., Zhu, H. J., ten Dijke, P. (2021). Cancer associated-fibroblast-derived exosomes in cancer progression. *Molecular Cancer, 20*. 10.1186/S12943-021-01463-Y 10.1186/s12943-021-01463-y PMC8638446 34852849

[200] Von Schulze A, Deng F (2020). A review on exosome-based cancer therapy. Journal of Cancer Metastasis and Treatment.

[201] Giacobino, C., Canta, M., Fornaguera, C., et al. (2021). Extracellular Vesicles and Their Current Role in Cancer Immunotherapy. *Cancers (Basel) 13*. 10.3390/CANCERS13092280 10.3390/cancers13092280 PMC8126043 34068657

[202] Lodestijn, S. C., Miedema, D. M., Lenos, K. J., et al. (2021). Marker-free lineage tracing reveals an environment-instructed clonogenic hierarchy in pancreatic cancer. *Cell Reports, 37*. 10.1016/J.CELREP.2021.109852 10.1016/j.celrep.2021.109852 34686335

[203] Sistigu A, Musella M, Galassi C (2020). Tuning Cancer Fate: Tumor Microenvironment’s Role in Cancer Stem Cell Quiescence and Reawakening. Frontiers in Immunology.

[204] Luo M, Li JF, Yang Q (2020). Stem cell quiescence and its clinical relevance. World Journal of Stem Cells.

[205] Chen K, Zhang C, Ling S (2021). The metabolic flexibility of quiescent CSC: Implications for chemotherapy resistance. Cell Death & Disease.

[206] Zhang H, Steed A, Co M, Chen X (2021). Cancer stem cells, epithelial-mesenchymal transition, ATP and their roles in drug resistance in cancer. Cancer Drug Resistance.

[207] Basu S, Dong Y, Kumar R (2022). Slow-cycling (dormant) cancer cells in therapy resistance, cancer relapse and metastasis. Seminars in Cancer Biology.

[208] De Angelis ML, Francescangeli F, La Torre F, Zeuner A (2019). Stem cell plasticity and dormancy in the development of cancer therapy resistance. Frontiers in Oncology.

[209] Batlle E, Clevers H (2017). Cancer stem cells revisited. Nature Medicine.

[210] Awasthi R, Roseblade A, Hansbro PM (2018). Nanoparticles in Cancer Treatment: Opportunities and Obstacles. Current Drug Targets.

[211] Reda A, Hosseiny S, El-Sherbiny IM (2019). Next-generation nanotheranostics targeting cancer stem cells. Nanomedicine.

[212] Mitra A, Mishra L, Li S (2015). EMT, CTCs and CSCs in tumor relapse and drug-resistance. Oncotarget.

[213] Seebacher, N. A., Krchniakova, M., Stacy, A. E., et al. (2021). Tumour Microenvironment Stress Promotes the Development of Drug Resistance. *Antioxidants 10*. 10.3390/ANTIOX10111801 10.3390/antiox10111801 PMC8615091 34829672

[214] Lee SH, Reed-Newman T, Anant S, Ramasamy TS (2020). Regulatory Role of Quiescence in the Biological Function of Cancer Stem Cells. Stem Cell Reviews and Reports.

[215] Holohan C, Van Schaeybroeck S, Longley DB, Johnston PG (2013). Cancer drug resistance: An evolving paradigm. Nature Reviews Cancer.

[216] López de Andrés J, Griñán-Lisón C, Jiménez G, Marchal JA (2020). Cancer stem cell secretome in the tumor microenvironment: A key point for an effective personalized cancer treatment. Journal of Hematology & Oncology.

[217] Yao Y, Zhou Y, Liu L (2020). Nanoparticle-Based Drug Delivery in Cancer Therapy and Its Role in Overcoming Drug Resistance. Frontiers in Molecular Biosciences.

[218] Yhee JY, Son S, Son S (2013). The EPR effect in cancer therapy. Cancer Targeted Drug Delivery: An Elusive Dream.

[219] Shi Y, van der Meel R, Chen X, Lammers T (2020). The EPR effect and beyond: Strategies to improve tumor targeting and cancer nanomedicine treatment efficacy. Theranostics.

[220] Senapati S, Mahanta AK, Kumar S, Maiti P (2018). Controlled drug delivery vehicles for cancer treatment and their performance. Signal Transduction and Targeted Therapy.

[221] Muzykantov VR (2013). Targeted Drug Delivery to Endothelial Adhesion Molecules. ISRN Vasc Med.

[222] Sakurai Y, Akita H, Harashima H (2019). Targeting tumor endothelial cells with nanoparticles. International Journal of Molecular Sciences.

[223] Bertrand N, Wu J, Xu X (2014). Cancer nanotechnology: The impact of passive and active targeting in the era of modern cancer biology. Advanced Drug Delivery Reviews.

[224] Mura S, Nicolas J, Couvreur P (2013). Stimuli-responsive nanocarriers for drug delivery. Nature Materials.

[225] Cheng CA, Deng T, Lin FC (2019). Supramolecular nanomachines as stimuli-responsive gatekeepers on mesoporous silica nanoparticles for antibiotic and cancer drug delivery. Theranostics.

[226] Thomas RG, Surendran SP, Jeong YY (2020). Tumor Microenvironment-Stimuli Responsive Nanoparticles for Anticancer Therapy. Frontiers in Molecular Biosciences.

[227] Rosenblum D, Joshi N, Tao W (2018). Progress and challenges towards targeted delivery of cancer therapeutics. Nature Communications.

[228] Dobrovolskaia MA, McNeil SE (2013). Understanding the correlation between in vitro and in vivo immunotoxicity tests for nanomedicines. Journal of Controlled Release.

[229] Urbán P, Liptrott NJ, Bremer S (2019). Overview of the blood compatibility of nanomedicines: A trend analysis of in vitro and in vivo studies. Wiley Interdisciplinary Reviews Nanomedicine Nanobiotechnology.

[230] Li Y, Fujita M, Boraschi D (2017). Endotoxin contamination in nanomaterials leads to the misinterpretation of immunosafety results. Frontiers in Immunology.

[231] Gerloff K, Landesmann B, Worth A (2017). The Adverse Outcome Pathway approach in nanotoxicology. Comput Toxicol.

[232] Sun B, Hyun H, Li L tao, Wang AZ (2020). Harnessing nanomedicine to overcome the immunosuppressive tumor microenvironment. Acta Pharmacologica Sinica.

[233] Liu Y, Guo J, Huang L (2020). Modulation of tumor microenvironment for immunotherapy: Focus on nanomaterial-based strategies. Theranostics.

[234] Zhang R, Liu T, Li W (2022). Tumor microenvironment-responsive BSA nanocarriers for combined chemo/chemodynamic cancer therapy. J Nanobiotechnology.

[235] Anselmo AC, Mitragotri S (2014). Cell-mediated delivery of nanoparticles: Taking advantage of circulatory cells to target nanoparticles. Journal of Controlled Release.

[236] Herrmann IK, Wood MJA, Fuhrmann G (2021). Extracellular vesicles as a next-generation drug delivery platform. Nature Nanotechnology.

[237] Maas SLN, Breakefield XO, Weaver AM (2017). Extracellular Vesicles: Unique Intercellular Delivery Vehicles. Trends in Cell Biology.

[238] Sanmartin MC, Borzone FR, Giorello MB (2022). Mesenchymal Stromal Cell-Derived Extracellular Vesicles as Biological Carriers for Drug Delivery in Cancer Therapy. Frontiers in Bioengineering and Biotechnology.

[239] Sedighi M, Zahedi Bialvaei A, Hamblin MR (2019). Therapeutic bacteria to combat cancer; current advances, challenges, and opportunities. Cancer Medicine.

[240] Gao, C., Wang, Q., Li, J., et al. (2022). In vivo hitchhiking of immune cells by intracellular self-assembly of bacteria-mimetic nanomedicine for targeted therapy of melanoma. *Science Advances, 8*. 10.1126/SCIADV.ABN1805 10.1126/sciadv.abn1805 PMC9094661 35544569

[241] Izci M, Maksoudian C, Manshian BB, Soenen SJ (2021). The Use of Alternative Strategies for Enhanced Nanoparticle Delivery to Solid Tumors. Chemical Reviews.

[242] Basak SK, Zinabadi A, Wu AW (2015). Liposome encapsulated curcumin-difluorinated (CDF) inhibits the growth of cisplatin resistant head and neck cancer stem cells. Oncotarget.

[243] Lu, B., Huang, X., Mo, J, Zhao, W. (2016). Drug delivery using nanoparticles for cancer stem-like cell targeting. *Frontiers in Pharmacology, 7*. 10.3389/fphar.2016.00084 10.3389/fphar.2016.00084 PMC4828437 27148051

[244] Chiacchiera F, Morey L, Mozzetta C (2020). Editorial: Epigenetic Regulation of Stem Cell Plasticity in Tissue Regeneration and Disease. Front Cell Dev Biol.

[245] Zhang, J., Arisha, A. H., Hua, J. (2021). Epigenetic regulation in stem cells. *Epigenetics and Reproductive Health,* 69–79. 10.1016/b978-0-12-819753-0.00004-0

[246] Takeda K, Mizushima T, Yokoyama Y (2018). Sox2 is associated with cancer stem-like properties in colorectal cancer. Science and Reports.

[247] Zhang S, Xiong X, Sun Y (2020). Functional characterization of SOX2 as an anticancer target. Signal Transduction and Targeted Therapy.

[248] Atlasi Y, Mowla SJ, Ziaee SAM, Bahrami AR (2007). OCT-4, an embryonic stem cell marker, is highly expressed in bladder cancer. International Journal of Cancer.

[249] Mohiuddin IS, Wei SJ, Kang MH (2020). Role of OCT4 in cancer stem-like cells and chemotherapy resistance. Biochimica et Biophysica Acta - Molecular Basis of Disease.

[250] Jeter CR, Yang T, Wang J (2015). Concise Review: NANOG in Cancer Stem Cells and Tumor Development: An Update and Outstanding Questions. Stem Cells.

[251] Gil-Kulik, P., Krzyżanowski, A., Dudzińska, E., et al. (2019). Potential involvement of BIRC5 in maintaining pluripotency and cell differentiation of human stem cells. *Oxidative Medicine and Cellular Longevity, 2019*. 10.1155/2019/8727925 10.1155/2019/8727925 PMC6350561 30774747

[252] Xu L, Yu W, Xiao H, Lin K (2021). BIRC5 is a prognostic biomarker associated with tumor immune cell infiltration. Science and Reports.

[253] Warrier NM, Agarwal P, Kumar P (2020). Emerging Importance of Survivin in Stem Cells and Cancer: The Development of New Cancer Therapeutics. Stem Cell Reviews and Reports.

[254] Neradil J, Veselska R (2015). Nestin as a marker of cancer stem cells. Cancer Science.

[255] Nagata T, Shimada Y, Sekine S (2014). Prognostic significance of NANOG and KLF4 for breast cancer. Breast Cancer.

[256] Rasti A, Mehrazma M, Madjd Z (2018). Co-expression of Cancer Stem Cell Markers OCT4 and NANOG Predicts Poor Prognosis in Renal Cell Carcinomas. Science and Reports.

[257] Lundberg IV, Edin S, Eklöf V (2016). SOX2 expression is associated with a cancer stem cell state and down-regulation of CDX2 in colorectal cancer. BMC Cancer.

[258] Schaefer T, Wang H, Mir P (2015). Molecular and functional interactions between AKT and SOX2 in breast carcinoma. Oncotarget.

[259] Warrier, N. M., Agarwal, P., Kumar, P. (2021). Integrative Analysis to Identify Genes Associated with Stemness and Immune Infiltration in Glioblastoma. *Cells, 10*. 10.3390/CELLS10102765 10.3390/cells10102765 PMC8534801 34685742

[260] Prager BC, Xie Q, Bao S, Rich JN (2019). Cancer Stem Cells: The Architects of the Tumor Ecosystem. Cell Stem Cell.

[261] Pai SG, Carneiro BA, Mota JM (2017). Wnt/beta-catenin pathway: Modulating anticancer immune response. Journal of Hematology & Oncology.

[262] Koni M, Pinnarò V, Brizzi MF (2020). The wnt signalling pathway: A tailored target in cancer. International Journal of Molecular Sciences.

[263] Mohammed MK, Shao C, Wang J (2016). Wnt/β-catenin signaling plays an ever-expanding role in stem cell self-renewal, tumorigenesis and cancer chemoresistance. Genes & Diseases.

[264] Luo J, Wang P, Wang R (2016). The Notch pathway promotes the cancer stem cell characteristics of CD90+ cells in hepatocellular carcinoma. Oncotarget.

[265] Venkatesh V, Nataraj R, Thangaraj GS (2018). Targeting notch signalling pathway of cancer stem cells. Stem Cell Investigation.

[266] Cochrane CR, Szczepny A, Watkins DN, Cain JE (2015). Hedgehog signaling in the maintenance of cancer stem cells. Cancers (Basel).

[267] Rinkenbaugh AL, Baldwin AS (2016). The NF-κB Pathway and Cancer Stem Cells. Cells.

[268] Xia Y, Shen S, Verma IM (2014). NF-κB, an active player in human cancers. Cancer Immunology Research.

[269] Kaltschmidt C, Banz-Jansen C, Benhidjeb T (2019). A role for NF-κB in organ specific cancer and cancer stem cells. Cancers (Basel).

[270] Hin Tang, J. J., Hao Thng, D. K., Lim, J. J., Toh, T. B. (2020). JAK/STAT signaling in hepatocellular carcinoma. *Hepatic Oncology, 7*. 10.2217/hep-2020-0001 10.2217/hep-2020-0001 PMC7137178 32273976

[271] Owen, K. L., Brockwell, N. K., Parker, B. S. (2019). Jak-stat signaling: A double-edged sword of immune regulation and cancer progression. *Cancers (Basel), 11*. 10.3390/cancers11122002 10.3390/cancers11122002 PMC6966445 31842362

[272] Thomas SJ, Snowden JA, Zeidler MP, Danson SJ (2015). The role of JAK/STAT signalling in the pathogenesis, prognosis and treatment of solid tumours. British Journal of Cancer.

[273] Brooks AJ, Putoczki T (2020). Jak-stat signalling pathway in cancer. Cancers (Basel).

[274] Herrera, S. C., Bach, E. A. (2019). JAK/STAT signaling in stem cells and regeneration: From drosophila to vertebrates. *Development, 146*. 10.1242/dev.167643 10.1242/dev.167643 PMC6361132 30696713

[275] Hill R, Wu H (2009). PTEN, stem cells, and cancer stem cells. Journal of Biological Chemistry.

[276] Cheng, H. S., Yip, Y. S., Lim, E. K. Y., et al. (2021). PPARs and tumor microenvironment: The emerging roles of the metabolic master regulators in tumor stromal-epithelial crosstalk and carcinogenesis. *Cancers (Basel), 13*. 10.3390/cancers13092153 10.3390/cancers13092153 PMC8125182 33946986

[277] Beyaz S, Yilmaz ÖH (2016). Molecular pathways: Dietary regulation of stemness and tumor initiation by the PPAR-d pathway. Clinical Cancer Research.

[278] Meyer-Hermann M (2018). Estimation of the cancer risk induced by therapies targeting stem cell replication and treatment recommendations. Science and Reports.

[279] Duan H, Liu Y, Gao Z, Huang W (2021). Recent advances in drug delivery systems for targeting cancer stem cells. Acta Pharm Sin B.

[280] Qin W, Huang G, Chen Z, Zhang Y (2017). Nanomaterials in targeting cancer stem cells for cancer therapy. Frontiers in Pharmacology.

[281] Yoshida GJ, Saya H (2016). Therapeutic strategies targeting cancer stem cells. Cancer Science.

[282] Song JH, Min SH, Kim SG (2022). Multi-functionalization Strategies Using Nanomaterials: A Review and Case Study in Sensing Applications. International Journal of Precision Engineering and Manufacturing-Green Technology.

[283] Angioletti-Uberti S (2017). Theory, simulations and the design of functionalized nanoparticles for biomedical applications: A Soft Matter Perspective. npj Computational Materials.

[284] Nano-SMART: Nanoparticles With MR Guided SBRT in Centrally Located Lung Tumors and Pancreatic Cancer - Full Text View - ClinicalTrials.gov. https://clinicaltrials.gov/ct2/show/NCT04789486?term=nano&cond=cancer&draw=2&rank=10. Accessed 6 Jun 2022

[285] Electroporation (NanoKnife) as Treatment for Advanced Pancreatic Cancer - Full Text View - ClinicalTrials.gov. https://clinicaltrials.gov/ct2/show/NCT02079623?term=nano&cond=cancer&draw=2&rank=11. Accessed 6 Jun 2022

[286] Abraxane Therapy in Patients With Pancreatic Cancer Who Failed First-Line Gemcitabine Therapy - Full Text View - ClinicalTrials.gov. https://clinicaltrials.gov/ct2/show/NCT00691054?term=nanoparticles&recrs=ade&cond=cancer&draw=2&rank=31. Accessed 6 Jun 2022

[287] Carboplatin and Nab-Paclitaxel With or Without Vorinostat in Treating Women With Newly Diagnosed Operable Breast Cancer - Full Text View - ClinicalTrials.gov. https://clinicaltrials.gov/ct2/show/NCT00616967?term=nanoparticles&recrs=ade&cond=cancer&draw=2&rank=37. Accessed 6 Jun 2022

[288] Paclitaxel Albumin-Stabilized Nanoparticle Formulation, Gemcitabine, and Bevacizumab in Treating Patients With Metastatic Breast Cancer - Full Text View - ClinicalTrials.gov. https://clinicaltrials.gov/ct2/show/study/NCT00662129?term=nanoparticles&recrs=ade&cond=cancer&draw=2&rank=3. Accessed 6 Jun 2022

[289] ABI-007 (Nab-Paclitaxel) and Gemcitabine in Treating Women With Metastatic Breast Cancer - Full Text View - ClinicalTrials.gov. https://clinicaltrials.gov/ct2/show/NCT00110084?term=nanoparticles&recrs=ade&cond=cancer&draw=2&rank=8. Accessed 6 Jun 2022

[290] S0800, Nab-Paclitaxel, Doxorubicin, Cyclophosphamide, and Pegfilgrastim With or Without Bevacizumab in Treating Women With Inflammatory or Locally Advanced Breast Cancer - Full Text View - ClinicalTrials.gov. https://clinicaltrials.gov/ct2/show/NCT00856492?term=nanoparticles&recrs=ade&cond=cancer&draw=2&rank=13. Accessed 6 Jun 2022

[291] Carboplatin and Paclitaxel Albumin-Stabilized Nanoparticle Formulation Followed by Radiation Therapy and Erlotinib in Treating Patients With Stage III Non-Small Cell Lung Cancer That Cannot Be Removed By Surgery - Full Text View - ClinicalTrials.gov. https://clinicaltrials.gov/ct2/show/NCT00553462?term=nanoparticles&recrs=ade&cond=cancer&draw=2&rank=19. Accessed 6 Jun 2022

[292] Paclitaxel Albumin-Stabilized Nanoparticle Formulation and Carboplatin in Treating Patients With Stage IIIB, Stage IV, or Recurrent Non-Small Cell Lung Cancer - Full Text View - ClinicalTrials.gov. https://clinicaltrials.gov/ct2/show/NCT00729612?term=nanoparticles&recrs=ade&cond=cancer&draw=2&rank=21. Accessed 6 Jun 2022

[293] A Phase 2 Study to Determine the Safety and Efficacy of BIND-014 (Docetaxel Nanoparticles for Injectable Suspension), Administered to Patients With Metastatic Castration-Resistant Prostate Cancer - Full Text View - ClinicalTrials.gov. https://clinicaltrials.gov/ct2/show/NCT01812746?term=nanoparticles&recrs=ade&cond=cancer&draw=2&rank=26. Accessed 6 Jun 2022

[294] Phase II NCT (Neoadjuvant Chemotherapy) w/ Weekly Abraxane in Combination With Carboplatin & Bevacizumab in Breast Cancer - Full Text View - ClinicalTrials.gov. https://clinicaltrials.gov/ct2/show/NCT00675259?term=nanoparticles&recrs=ade&cond=cancer&draw=2&rank=29. Accessed 6 Jun 2022

[295] Paclitaxel Albumin-Stabilized Nanoparticle Formulation in Treating Patients With Previously Treated Advanced Non-small Cell Lung Cancer - Full Text View - ClinicalTrials.gov. https://clinicaltrials.gov/ct2/show/NCT01620190?term=nanoparticles&recrs=ade&cond=cancer&draw=2&rank=30. Accessed 6 Jun 2022

[296] Fiorillo M, Verre AF, Iliut M (2015). Graphene oxide selectively targets cancer stem cells, across multiple tumor types: Implications for non-toxic cancer treatment, via “differentiation-based nano-therapy”. Oncotarget.

[297] Choi YJ, Gurunathan S, Kim JH (2018). Graphene oxide-silver nanocomposite enhances cytotoxic and apoptotic potential of salinomycin in human ovarian cancer stem cells (OvCSCs): A novel approach for cancer therapy. International Journal of Molecular Sciences.

[298] Knauer N, Arkhipova V, Li G (2022). In Vitro Validation of the Therapeutic Potential of Dendrimer-Based Nanoformulations against Tumor Stem Cells. International Journal of Molecular Sciences.

[299] Yao HJ, Zhang YG, Sun L, Liu Y (2014). The effect of hyaluronic acid functionalized carbon nanotubes loaded with salinomycin on gastric cancer stem cells. Biomaterials.

[300] Al Faraj A, Shaik AS, Ratemi E, Halwani R (2016). Combination of drug-conjugated SWCNT nanocarriers for efficient therapy of cancer stem cells in a breast cancer animal model. Journal of Controlled Release.

[301] Yi Y, Kim HJ, Zheng M (2019). Glucose-linked sub-50-nm unimer polyion complex-assembled gold nanoparticles for targeted siRNA delivery to glucose transporter 1-overexpressing breast cancer stem-like cells. Journal of Controlled Release.

[302] Zhao Y, Zhao W, Lim YC, Liu T (2019). Salinomycin-Loaded Gold Nanoparticles for Treating Cancer Stem Cells by Ferroptosis-Induced Cell Death. Molecular Pharmaceutics.

[303] Liang S, Li C, Zhang C (2015). CD44v6 monoclonal antibody-conjugated gold nanostars for targeted photoacoustic imaging and plasmonic photothermal therapy of gastric cancer stem-like cells. Theranostics.

[304] Poonaki, E., Nickel, A. C., Ardestani, M. S., et al. (2022). CD133-Functionalized Gold Nanoparticles as a Carrier Platform for Telaglenastat (CB-839) against Tumor Stem Cells. *International Journal of Molecular Sciences, 23*. 10.3390/IJMS23105479/S1 10.3390/ijms23105479 PMC9141725 35628289

[305] Locatelli E, Li Y, Monaco I (2019). A novel theranostic gold nanorods- and adriamycin-loaded micelle for EpCA M targeting, laser ablation, and photoacoustic imaging of cancer stem cells in hepatocellular carcinoma. International Journal of Nanomedicine.

[306] Liu Y, Yang M, Zhang J (2016). Human Induced Pluripotent Stem Cells for Tumor Targeted Delivery of Gold Nanorods and Enhanced Photothermal Therapy. ACS Nano.

[307] Zhou J, Sun M, Jin S (2019). Combined using of paclitaxel and salinomycin active targeting nanostructured lipid carriers against non-small cell lung cancer and cancer stem cells. Drug Delivery.

[308] Arthur P, Patel N, Surapaneni SK (2020). Targeting lung cancer stem cells using combination of Tel and Docetaxel liposomes in 3D cultures and tumor xenografts. Toxicology and Applied Pharmacology.

[309] Wang, Z., Sun, M., Li, W., et al. (2020). A Novel CD133- and EpCAM-Targeted Liposome With Redox-Responsive Properties Capable of Synergistically Eliminating Liver Cancer Stem Cells. *Frontiers in Chemistry, 8*. 10.3389/FCHEM.2020.00649 10.3389/fchem.2020.00649 PMC7431664 32850663

[310] Ke XY, Lin Ng VW, Gao SJ (2014). Co-delivery of thioridazine and doxorubicin using polymeric micelles for targeting both cancer cells and cancer stem cells. Biomaterials.

[311] Li L, Cui D, Ye L (2017). Codelivery of salinomycin and docetaxel using poly(d, l-lactic-co-glycolic acid)-poly(ethylene glycol) nanoparticles to target both gastric cancer cells and cancer stem cells. Anti-Cancer Drugs.

[312] Xu CF, Liu Y, Shen S (2015). Targeting glucose uptake with siRNA-based nanomedicine for cancer therapy. Biomaterials.

[313] Espinosa-Cano E, Huerta-Madroñal M, Cámara-Sánchez P (2021). Hyaluronic acid (HA)-coated naproxen-nanoparticles selectively target breast cancer stem cells through COX-independent pathways. Materials Science and Engineering C.

[314] Moro, M., Fortunato, O., Bertolini, G., et al. (2022). MiR-486-5p Targets CD133+ Lung Cancer Stem Cells through the p85/AKT Pathway. *Pharmaceuticals, 15*. 10.3390/PH15030297/S1 10.3390/ph15030297 PMC8951736 35337095

[315] Pang L, Huang X, Zhu L (2022). Targeted killing of CD133 + lung cancer stem cells using paclitaxel-loaded PLGA-PEG nanoparticles with CD133 aptamers. Nan Fang Yi Ke Da Xue Xue Bao.

[316] Zhao Y, Wang K, Zheng Y (2021). Co-delivery of Salinomycin and Curcumin for Cancer Stem Cell Treatment by Inhibition of Cell Proliferation, Cell Cycle Arrest, and Epithelial-Mesenchymal Transition. Frontiers in Chemistry.

[317] Jiang J, Li H, Qaed E (2018). Salinomycin, as an autophagy modulator - A new avenue to anticancer: A review. Journal of Experimental & Clinical Cancer Research.

[318] Tefas LR, Barbălată C, Tefas C, Tomuță I (2021). Salinomycin-based drug delivery systems: Overcoming the hurdles in cancer therapy. Pharmaceutics.

[319] Kim KY, Il PK, Kim SH (2017). Inhibition of Autophagy Promotes Salinomycin-Induced Apoptosis via Reactive Oxygen Species-Mediated PI3K/AKT/mTOR and ERK/p38 MAPK-Dependent Signaling in Human Prostate Cancer Cells. International Journal of Molecular Sciences.

[320] Wang H, Zhang H, Zhu Y (2021). Anticancer Mechanisms of Salinomycin in Breast Cancer and Its Clinical Applications. Frontiers in Oncology.

[321] Urbaniak A, Reed MR, Fil D (2021). Single and double modified salinomycin analogs target stem-like cells in 2D and 3D breast cancer models. Biomedicine & Pharmacotherapy.

[322] Li B, Wu J, Tang L (2022). Synthesis and anti-tumor activity evaluation of salinomycin C20- O -alkyl/benzyl oxime derivatives. Organic & Biomolecular Chemistry.

[323] Czerwonka D, Mü S, Cañ T (2022). Expeditive Synthesis of Potent C20-epi-Amino Derivatives of Salinomycin against Cancer Stem-Like Cells. ACS Organic & Inorganic Au.

[324] Li R, Guo N, Fu L, Miao Y (2022). A Feasible Strategy of Fabricating Redox-Responsive Polymeric Salinomycin Small Molecule Prodrug Delivery for Liver Cancer Therapy. Journal of Cluster Science.

[325] Thomas OS, Weber W (2019). Overcoming Physiological Barriers to Nanoparticle Delivery—Are We There Yet?. Front Bioeng Biotechnol.

[326] Liang DS, Liu J, Peng TX (2018). Vitamin E-based redox-sensitive salinomycin prodrug-nanosystem with paclitaxel loaded for cancer targeted and combined chemotherapy. Colloids Surfaces B Biointerfaces.

[327] Wang J, Zhuo J, Tao Y (2020). <p>Salinomycin-Loaded Small-Molecule Nanoprodrugs Enhance Anticancer Activity in Hepatocellular Carcinoma</p>. International Journal of Nanomedicine.

[328] Sun W, Luo JD, Jiang H, Duan DD (2018). Tumor exosomes: A double-edged sword in cancer therapy. Acta Pharmacologica Sinica.

[329] Aqil, F., Gupta, R. C. (2022). Exosomes in Cancer Therapy. *Cancers (Basel), 14*. 10.3390/CANCERS14030500 10.3390/cancers14030500 PMC8833672 35158768

[330] Ståhl AL, Johansson K, Mossberg M (2019). Exosomes and microvesicles in normal physiology, pathophysiology, and renal diseases. Pediatric Nephrology (Berlin, Germany).

[331] Lee Chung B, Toth MJ, Kamaly N (2015). Nanomedicines for endothelial disorders. Nano Today.

[332] Du Y, Wang S, Zhang M (2021). Cells-Based Drug Delivery for Cancer Applications. Nanoscale Research Letters.

[333] Garcia-Heredia JM, Lucena-Cacace A, Verdugo-Sivianes EM (2017). The cargo protein MAP17 (PDZK1IP1) regulates the cancer stem cell pool activating the Notch pathway by abducting NUMB. Clinical Cancer Research.

[334] Pinho S, Macedo MH, Rebelo C (2018). Stem cells as vehicles and targets of nanoparticles. Drug Discovery Today.

[335] Lenna S, Bellotti C, Duchi S (2020). Mesenchymal stromal cells mediated delivery of photoactive nanoparticles inhibits osteosarcoma growth in vitro and in a murine in vivo ectopic model. Journal of Experimental & Clinical Cancer Research.

[336] Mercer-Smith AR, Findlay IA, Bomba HN, Hingtgen SD (2021). Intravenously Infused Stem Cells for Cancer Treatment. Stem Cell Rev Reports.

[337] Hassanzadeh A, Altajer AH, Rahman HS (2021). Mesenchymal Stem/Stromal Cell-Based Delivery: A Rapidly Evolving Strategy for Cancer Therapy. Front Cell Dev Biol.

